# Taxing for healthier beginnings: The impact of a major tobacco tax hike on birth weight in Mexico

**DOI:** 10.1016/j.ssmph.2025.101851

**Published:** 2025-08-26

**Authors:** Francisco Beltran-Silva, Rodrigo Aranda

**Affiliations:** a Department of Economics, California State University-Northridge, 18111 Nordhoff Street, Northridge, CA 91330, United States of America; b Department of Economics, Western Michigan University, United States of America

**Keywords:** Birth outcomes, Cigarettes, Fetal health, Taxation policy, Tobacco

## Abstract

Tobacco consumption during pregnancy poses significant risks to maternal and neonatal health. In 2011, Mexico implemented a large nationwide increase in tobacco excise taxes. Because the policy was applied uniformly across the country and detailed smoking data are limited, identifying causal effects is particularly challenging. Using comprehensive vital statistics records on all singleton live births in Mexico, we apply a regression discontinuity in time design to evaluate the short-term impact of a 250% increase in the excise tax on tobacco products on newborn health outcomes. Our findings provide evidence of moderate short-term increases in birth weight after the tax hike. Although the effects diminish over time and show sensitivity to model specification, they may indicate potential long-term public health benefits. This study provides new evidence on the effects of nationwide tobacco tax increases on birth outcomes in middle-income countries.

## Highlights

• Mexico’s 2011 large nationwide tobacco tax led to modest birth weight gains.
• Regression discontinuity in time used to estimate short-term effects.
• Health benefits faded in the months after policy implementation.
• Study adds new evidence on tobacco policy effects in middle-income countries.

## 1 Introduction

Tobacco smoking and its associated health risks are a major public health concern that contributes to a substantial burden of morbidity and mortality around the world. Additionally, tobacco is responsible for approximately 8 million deaths per year. Of these deaths, around 7 million are attributed to direct tobacco use and an additional 1.2 million are the result of exposure to second-hand smoke.^1^

Smoking rates in Mexico remain high despite years of anti-smoking policies. In 2023, the prevalence of smoking among people 20 years and older was approximately 19.6%. In addition, men had a higher smoking rate (28.4%) compared to women (11.6%) (ENSANUT, 2023). In 2021, approximately 9.7% of deaths in Mexico were attributed to smoking, resulting in approximately 63.2 thousand deaths (IECS, 2020).

Smoking during pregnancy increases the risk that an infant will be born with low birth weight (Hamilton, 2001, Shiono and Behrman, 1995, Suarez et al., 2008). In addition to low birth weight, maternal smoking is associated with an increased risk of complications such as miscarriage, preterm birth, congenital abnormalities, and respiratory disorders. Low birth weight is also a major health concern, as it is also associated with a variety of chronic conditions during adulthood (Bianchi and Restrepo, 2022, Risnes et al., 2011). Moreover, smoking seems to be a relevant mechanism in the production of socioeconomic disparities in health at birth (Panico & Tô, 2023).

Pregnant women are a group at increased risk of tobacco exposure that has not received enough attention when examining the impact of tobacco policies in Mexico. Although Mexico has a relatively low overall self-reported incidence of smoking among pregnant women 8.0% (Sánchez-Zamorano et al., 2004), the incidence remains high in the population in general. For example, a significant percentage (16.1%) of nonsmoking women are exposed to second-hand smoke at home, while exposure rates are even higher in restaurants (31.5%), public transportation (26.2%) and workplaces (16.6%) (CONADIC, 2017). This pervasive exposure is particularly concerning given the evidence that second-hand smoke increases the risk of low birth weight by more than twofold (Handriani et al., 2022).

Despite the well-documented health risks of prenatal tobacco exposure and a growing body of research on the health benefits of tobacco control, less is known about how such tobacco taxation affects birth outcomes, particularly in low- and middle-income countries (Akter et al., 2023). Research in Mexico has largely centered on patterns of tobacco use and the broader social consequences of tobacco taxation. Atondo-García et al., 2025, Jiménez-Ruiz et al., 2008, Olivera-Chávez et al., 2010, Reynales-Shigematsu et al., 2020, Sánchez-Zamorano et al., 2004, Zavala-Arciniega et al., 2020. Limited access to high-frequency behavioral data, combined with the absence of regional variation in policy implementation, has made it challenging to rigorously assess the health effects of national tobacco tax reforms.

In this paper, we estimate the short-term effects of a major tobacco tax on birth outcomes, focusing on birth weight. To do so, we consider one of the largest increases in tobacco taxes in Mexico in recent history. Implemented in January 2011, the increase in the tobacco quota went from 0.10 to 0.35 pesos per cigarette, a 250% increase. We use administrative data on birth records derived from Mexico’s vital statistics data. For context, this tax increased the price of a 20-cigarette pack by 7 pesos. Equivalent tax hikes were applied to other smoked tobacco products according to their weight. This tax increase was equivalent to a 36 percent price increase for the most popular brands of cigarettes that cost between 25 and 38 pesos before the tax increase (Reynales-Shigematsu et al., 2020).

Our focus on birth weight as the primary outcome of interest is motivated by its status as a well-established indicator of fetal development and its consistent association with maternal smoking behaviors and second-hand smoke (Bernstein et al., 2005, Hamilton, 2001, Handriani et al., 2022, Suarez et al., 2008). Moreover, it is more accurate than variables like gestational age as it is measured with standard equipment at delivery (Balchin et al., 2004, Rozendaal et al., 2012, Snell et al., 1992). While birth weight remains central to our analysis, we also assess whether the tax affected other dimensions of newborn health, including respiratory complications, gestational duration, extreme cases of low birth weight, neonatal length, and congenital anomalies in line with related research (Hackshaw et al., 2011, Spindel and McEvoy, 2016, Wen et al., 1990).

Since the policy was implemented nationwide at the same time, isolating a causal relationship is particularly challenging due to the absence of a natural untreated comparison group. To address this, we adopt a regression discontinuity in time approach. This identification strategy leverages the precise timing of the tobacco tax increase as a cut-off point. This design allows us to compare birth outcomes just before and after the policy change under the assumption that, in the absence of the tax, birth outcomes would have followed a similar trend over time. Although we cannot directly observe mother smoking behavior and their second-hand smoke exposure, this strategy provides a plausibly causal estimate of the short-term impact of tobacco taxation on birth outcomes.

We find that Mexico’s 2011 tobacco tax increase was associated with moderate but statistically significant improvements in birth weight of approximately 10 g. These effects, although short-lived, suggest that fiscal policy can have measurable health benefits in middle-income country settings. Furthermore, we find no effects of the increase in the tobacco tax on infant respiratory outcomes, gestational length, or congenital anomalies.

This article adds to the already existing body of knowledge on tobacco taxes and birth outcomes in several important ways. First, it addresses a significant evidence gap in the evaluation of Mexico’s tobacco control policies-largely driven by limitations in behavioral data associated with smoking-using a quasi experimental research design and administrative birth records. To our knowledge, this is the first national-level analysis of the impact of the 2011 tobacco tax increase on birth outcomes in Mexico. Second, it broadens existing research by examining the effects of large-scale tobacco taxation in the context of middle-income countries, thus expanding the insights from previous studies conducted in high-income settings (Akter et al., 2023, Faber et al., 2017, Lien and Evans, 2005).

## 2 Maternal smoking and tobacco taxation in Mexico

Smoking-related medical care accounts for about 4.3% of the annual expenditures of the Mexican Institute of Social Security (IMSS), which covers 49% of the insured population (Reynales-Shigematsu et al., 2006, Rivera-Hernandez et al., 2016). In Mexico, the incidence of active smoking during pregnancy is estimated to be 8.0% (Sánchez-Zamorano et al., 2004). Globally, smoking during pregnancy remains a public health concern, with an estimated 12% of pregnant women smoking worldwide (Restivo et al., 2020). Among pregnant women in low- and middle-income countries, prevalence tends to be lower, with around 2.1% reporting active smoking. Yet this is far below the 24.4% who report exposure to second-hand smoke at home (Yang et al., 2022). These global disparities are also reflected in Mexico, where a significant number of non-smoking women in Mexico are exposed to second-hand smoke in various settings. Approximately 16.1% experience exposure at home, while many also encounter it at work (16.6%), restaurants (31.5%), and public transportation (26.2%) (CONADIC, 2017). Even among women who have never smoked themselves, 14.4% report being exposed to second-hand smoke at home. These figures reflect global trends, where approximately one-third of women worldwide were exposed to second-hand smoke in 2016 (Drope et al., 2018).

In response to the health and economic burdens caused by smoking, Mexico has adopted a variety of tobacco control policies (Reynales-Shigematsu et al., 2020). In 2004 Mexico signed the Framework Convention on Tobacco Control (FCTC) of the World Health Organization (WHO). To ensure adherence and implementation of the policies outlined by FCTC, Mexico established a permanent platform known as the Office of Tobacco Control (OTC) in 2008. In the same year, smoke-free legislation was enacted in bars, restaurants, and workplaces. In 2009, cigarettes ads were banned from television and radio. Starting in 2010, several waves of pictorial warnings on cigarette packages content were released. A detailed chronology of these and other measures can be found in Reynales-Shigematsu et al. (2020).

Before the introduction of any specific taxes, tobacco products used for smoking were only taxed through the general Value Added Tax at a rate of 15%. In November 2009, the previously mentioned Value Added Tax was increased from 15% to 16% affecting all goods, including cigarettes. However, on 1 January 2010, the government also introduced a tax in the form of a quota of $0.10 per cigarette to imported and nationally produced products. On January 1, 2011, there was a major increase in the quota from $0.10 to $0.35 pesos per cigarette. In subsequent years, the quota per cigarette and other tobacco products has been updated to account for inflation. Table 1 shows the dates of the key changes in the excise taxes on cigarettes in Mexico. The increase in the quota per cigarette and other tobacco products used for smoking that occurred on January 1, 2011 has been one of the largest and most unprecedented changes in tobacco taxes in Mexico in recent years as it went from $0.10 to $0.35 pesos per cigarette more than tripling the tax. The change was equivalent to an increase of 7 pesos per pack in 20 cigarette packages. The price of some brands increased more than others, the price of the pack of one of the most popular brands, for example, increased 36% with this change, from 28 MXN (2.30 USD) to 38 MXN (3.12 USD) (Reynales-Shigematsu et al., 2020).

Taken together, it can be assumed that the tobacco policies implemented in recent years have shown a degree of effectiveness in reducing smoking prevalence. The prevalence of smoking in the population appears to have decreased from 21.5% to 19.0% between 2002 and 2016, (Zavala-Arciniega et al., 2020). There has been a notable decline in the percentage of individuals who smoke on a daily basis, dropping from 24.0% to 11.9%. The average number of cigarettes smoked by adults also decreased, from about 7.5 in 2006 to 6.3 in 2012. However, the prevalence of smoking among adult tobacco users showed little variation, remaining at 19% in 2006 and slightly increasing to 19.9% in 2012. Furthermore, when comparing Mexico data in 2009 and 2015 data, it can be observed that there were slight decreases in self-reported exposure to second-hand smoke in government buildings (17.0% to 14.1%), restaurants (29.6% to 24.6%), and bars and nightclubs (81.2% to 72.7%) (Reynales-Shigematsu et al., 2020). Rigorous empirical research evaluating the causal impact of specific tobacco control measures—such as large tax increases—remains scarce, in large part due to the limited availability of high-frequency and individual-level data on smoking behaviors and exposures.

**Table 1:** Changes in excise taxes on cigarettes in Mexico.

Announcement date	Introduction date	Description
November 27, 2009	January 1st of 2010	The tax consisted of a quota of $0.10 per cigarette. This tax was equivalent to 2 pesos per pack on 20 cigarette packages.

November 19, 2010	January 1st of 2011	The quota increased from $0.10 to $0.35 pesos per cigarette sold. This tax was equivalent to 7 pesos per pack on 20 cigarette packages.

December 24, 2019	January 1st, 2020	The quota increased from $0.35 to $0.4944 pesos per cigarette sold. This tax was equivalent to $9.88 pesos per pack on 20 cigarette packages. The increment was enacted as an adjustment for inflation from previous years.

Notes: Own elaboration derived from the Special Tax on Production and Services General Law (Ley del Impuesto Especial Sobre Producción y Servicios (IEPS)) spanning various years, with analysis based on the provided legislation. The law considered the weight of a cigarette to be 0.75 g of tobacco, including the weight of any other substances with which the tobacco might be mixed but excluding filter or any other substance not containing tobacco with which the cigarettes are wrapped. For tobacco products different from a standard cigarette of 0.75 g, for example, handmade cigars, the order set a tax per unit to the result of dividing the weight of the tobacco product by 0.75 g. The government tax applied to nationally produced or imported products. For reference the taxation measure implemented amounted to 0.80 pesos per pack of cigarettes in 2010, 1.20 pesos in 2011, 1.60 pesos in 2012, and finally increased to 2.00 pesos in 2013.

## 3 Methods

### 3.1 Data

This study uses vital statistics records of all singleton live births in Mexico. These publicly accessible data include all legally reported births and are maintained by the Mexico Ministry of Health (Secretaría de Salud, 2019). With an annual count of approximately 2 million births, this information facilitates a precise estimate of national effects. The main sample consists of women aged 18 to 44 years with singleton deliveries. We exclude cases with extreme birth weight values (below 500 g and above 7000 g). These extreme cases likely reflect data entry errors.^2^ Our analysis focuses on observations surrounding the 2011 implementation of the policy, although we used data spanning January 1, 2008, to December 31, 2019, throughout our analysis. We obtained the exact dates of implementation of the tax increase from the Federation Official Diary (DOF (Official Gazette of the Federation - Diario Oficial de la Federación), 2010). We matched these dates to specific birth-related dates using the vital statistics data.

### 3.2 Outcomes of interest

Our main outcome of interest is the birth weight in grams. Birth weight serves as a sensitive and well-established indicator of fetal development, with previous studies demonstrating its responsiveness to maternal smoking behaviors and second-hand smoke (Bernstein et al., 2005, Hamilton, 2001, Handriani et al., 2022, Suarez et al., 2008). It is also among the most reliably measured outcomes in administrative birth records, as it is recorded at delivery using standardized procedures. In contrast, other outcomes—such as respiratory complications, gestational length, or congenital anomalies—are more susceptible to underreporting, misclassification, or imprecise measurement (Balchin et al., 2004, Rozendaal et al., 2012, Snell et al., 1992). Moreover, low birth weight is linked to negative health outcomes such as infant mortality and chronic diseases in adulthood (Bianchi and Restrepo, 2022, Risnes et al., 2011).

To assess the effects of the tax increase on birth weight, we examine four different outcome measures: infant birth weight in grams, an indicator for low birth weight (less than 2500 g), normal birth weight (in between 2500 and 4000 g) and fetal macrosomia (over 4000 g). In addition to birth weight outcomes, we delved into a comprehensive analysis of supplementary outcome measures. These include respiratory issues, such as the Silverman score and any recorded respiratory complications, as well as variables related to the gestational length of the pregnancy, such as occurrences of postterm and preterm births. We also considered other relevant outcomes, such as having very low birth weight and extreme low birth weight, infant length, and the presence of congenital anomalies. Broadening our investigation to encompass this set of outcomes helps us build a more complete picture of how the tax may have influenced newborn health.

### 3.3 Analytical strategy

Since the policy was implemented uniformly throughout the country, identifying a causal effect is especially challenging in the absence of a natural comparison group. Another key challenge is that, unlike other countries, Mexican birth records do not include information on smoking behavior, which prevents us from directly measuring how the tax affected smoking among pregnant women. This contrasts to studies such as (Lien & Evans, 2005), which can use tax variation as an instrument to estimate the causal effect of maternal smoking on birth outcomes.

There are also no consistent annual data on tobacco or cigarette consumption in Mexico, further limiting our ability to trace a clear pathway from taxation to improved health outcomes. For example, data from the Mexican National Health Survey (ENSA) and the Mexican National Health and Nutrition Survey (ENSANUT) show that daily smoking among adults fell from 13.3% in 2006 to 11.8% in 2012 (Guerrero-López et al., 2013). However, the long intervals between survey waves make it difficult to attribute these changes specifically to the 2011 tax increase, as they may also reflect other policy efforts or broader trends. Unfortunately, we also lack annual data on smoking behavior among women or pregnant women which limits our ability to estimate how maternal or second-hand smoke exposure changed after the tax increase. While 8.0% of pregnant women in Mexico report smoking (Sánchez-Zamorano et al., 2004), many more are exposed to second-hand smoke, ranging from 16.1% at home to more than 30% in settings such as restaurants, public transportation, and workplaces (CONADIC, 2017). Despite data limitations, these figures suggest that a considerably large share of pregnant women may benefit from tobacco control policies.

**Fig. 1:**
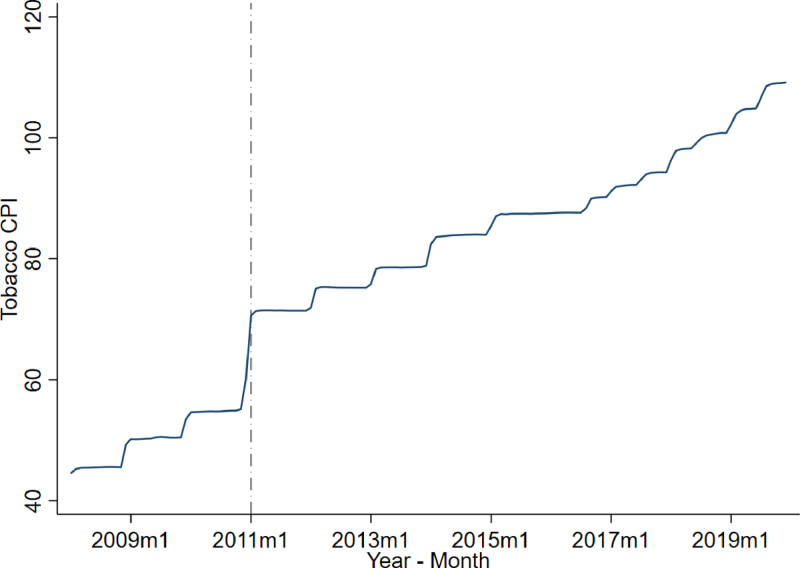
Evolution of tobacco prices in Mexico. *Notes:* This graph shows the monthly evolution of the Tobacco Consumer Price Index in Mexico obtained from INEGI (National Institute of Statistics and Geography — Instituto Nacional de Estadística y Geografía) from 2008 to 2019. The vertical line represents January 1st, 2011, which marks the date of the largest increase in the excise tobacco tax.

Given these data limitations, we rely on indirect evidence to assess the possible impact of the 2011 tax increase on smoking behavior and related health outcomes. The research on cigarette price elasticity in Mexico estimates that a 10% increase in cigarette prices could lead to a 2.5 to 6.4% decrease in consumption (Jiménez-Ruiz et al., 2008, Olivera-Chávez et al., 2010). This implies that if the 2011 tax increase significantly raised prices, it likely led to a significant decline in smoking. This reduction would plausibly extend to pregnant women and also to those in close proximity, such as family members, reducing both direct and second-hand smoke exposure during pregnancy. Fig. 1 supports this interpretation by showing a clear and immediate increase in the consumer price index of cigarettes beginning in January 2011, precisely coinciding with the implementation of the tax hike. The observed pattern supports the hypothesis that the 2011 tax increase affected birth outcomes by incentivizing pregnant women to reduce or stop smoking, as well as by decreasing exposure to second-hand smoke. Dasgupta et al. (2022) provides support to the causal link between prenatal tobacco exposure and birth outcomes using variation from changes in federal and state-level tobacco tax rates as an instrument.

The lack of behavioral data limits our ability to disentangle relative contributions. Our hypothesis aligns with extensive evidence showing that tobacco taxes are an effective policy tool to reduce smoking, including during pregnancy, and are consistently associated with improved birth outcomes, especially among socioeconomically disadvantaged populations (Markowitz et al., 2013, Radó et al., 2022, WHO, 2008). The estimated effects obtained from our analysis should therefore be understood as reflecting the combined effect of reduced direct and indirect tobacco exposure during pregnancy.

Our main assumption is that, after taking seasonal and regional elements into consideration, any sudden alterations in birth results after and around January 1, 2011 are linked to the implementation of tobacco taxes. Our empirical strategy leverages the sharp change in prices observed when the tax change came into effect. Thus, we assume that in the absence of the tax modification, birth weight and other outcomes at birth would have exhibited a consistent and uninterrupted trend. We opt for a sharp discontinuity design because every woman in the sample experiences the tax increase. The decision rule determining exposure to the intervention and the January 1, 2011 cut-off value are publicly known. This research strategy has been frequently applied across various disciplines, such as economics and finance; see, for example, Aguilar et al., 2021, Byker, 2016. Our approach can be considered as an event study or as a regression discontinuity design that uses time as a running variable.

### 3.4 Regression discontinuity approach

We use the date at which the third trimester of pregnancy begins as our preferred cut-off point for exposure in our analysis. Using the dates of birth, we compare infants entering the third trimester just after implementation with those entering the third trimester just before the tax increase. This choice aligns with a similar study that analyzes the impact of smoke-free legislation on birth outcomes using a regression discontinuity approach (Bakolis et al., 2016). The American Pregnancy Association notes that the third trimester is critical for fetal growth, while the American Lung Association reports that lung development typically completes around week 37.

In line with (Calonico et al., 2014, Hausman and Rapson, 2018), we use an augmented local linear procedure. This two-step process uses the full sample to identify significant regressors. Subsequently, it estimates the conditioned second stage using a narrower sample bandwidth, eliminating the necessity for global polynomials over the whole sample. In the first stage, we estimate Eq. (1) by ordinary least squares to obtain the corresponding residuals including all observations available from 2008 to 2019.

(1) $$Y_{it}=\gamma_{w}+\lambda_{m}+\eta_{a}+{\overset{˜}{Y}}_{it}$$

Where $Y_{it}$ denotes the outcome of interest measuring the health of the newborn, ${\overset{˜}{Y}}_{it}$ represents the residual term, i stands for the individual birth which is the unit of observation, m stands for municipality, and t stands for a specific date. Birth weight exhibits a cyclical pattern, with recurring trends of increased birth weight during the year. Such seasonal fluctuations tied to maternal cycles are accounted for by incorporating week-of-the-year fixed effects $\gamma_{w}$. Geographic discrepancies are captured by incorporating municipality fixed effects associated with the mother’s residence at delivery $\lambda_{m}$. In addition, we include year fixed effects, $\eta_{a}$, to capture any systematic variations associated with changes in the way information was recorded, broader economic trends, and other unobserved time-varying factors. Fertility, for example, increased considerably in 2010 and 2011 before beginning to decrease in 2012 (Mie y Terán Rocha & García Guerrero, 2019). Alternatively, we utilize polynomials in time of different order to capture these variations, f(t).

To do the estimation, we first partial out week-of-the-year when each birth occurs, the mother’s reported municipality of residence at delivery and year fixed effects. This process yields residuals that remove week-level seasonality, time-varying factors within a given year, and location-specific components.

Over time, the certificates used to capture information about birth records have undergone changes as new formats of birth certificates emerged. Some years, more than one format was used. To account for systematic changes in data collection associated with these formats, we control for the format version utilized to record each individual birth record. Four format versions were used between 2008 and 2019: the pre-2010 format, the 2010 format, the 2015 format, and the most recent 2016 format. The adoption of new formats was not uniform. For example, in 2011, a significant portion of births were registered using the format before 2010, while some were registered using the 2010 format.

Furthermore, to include a metric of socioeconomic conditions over time and between states, we control for monthly unemployment rates at the state level $U_{st}$. This mirrors the common approach in the literature on air pollution policy evaluation, which, in addition to adjusting for seasonality using effects of the month or day of the week, also considers weather conditions such as temperature, rainfall, and snowfall (Hausman & Rapson, 2018).

The second stage of the augmented local linear procedure consists of using the residuals obtained from Eq. (1) in Eq. (2), where $Y_{it}\left(1\right)$ and $Y_{it}\left(0\right)$ denote the potential outcomes with and without the tax where m stands for municipality and t stands for an specific date. Let $\bar{t}$ denote January 1st, 2011. The birth i is then treated at time t when $t\geq \bar{t}$.

(2) $${\overset{ˆ}{Y}}_{it}=\left\{\begin{array}{c} {\overset{ˆ}{Y}}_{it}\left(0\right)\text{if}t<\bar{t}\\ {\overset{ˆ}{Y}}_{it}\left(1\right)\text{if}t\geq \bar{t} \end{array}\right)$$

Our interest is to estimate the average treatment effect of the tax close to the enactment date $\tau =E\left[{\overset{ˆ}{Y}}_{it}\left(1\right)-{\overset{ˆ}{Y}}_{}it\left(0\right)|t=\bar{t}\right]$. The estimator for τ is constructed using a kernel-based local polynomial on either side of the threshold with different bandwidths. As the tax reform analyzed lacks cross-sectional variation in treatment status, our focus is predominantly on short-term effects.

The regression discontinuity design assigns the exposure of interest based on the value of a continuously measured random variable above and below a threshold. We use the time between the date at entering the third trimester of pregnancy, week 28, and the cut-off date, which is the date the tax was introduced, as the threshold. These thresholds are obtained based on the gestational age in weeks reported in the birth records in relation to the date of birth. This threshold is calculated by counting back from gestational age in weeks at birth. Exposure is assumed to be independent of the policy’s influence. The assignment variable is measured in days to ensure its continuity near the cut-off point.

We adopt the date at which the third trimester of pregnancy begins as our preferred cut-off point for exposure in our analysis. Infants entering the third trimester immediately before the hike would have partial exposure, while those entering the third trimester after would have increased exposure. Given our identification strategy, our comparison primarily delves into the impact of increasing pregnancy exposure with focus on the short-term effects. We alternatively assign treatment based on entry into the first, second, and third trimesters for additional insights. Assigning treatment based on the start of the first trimester, for example, facilitates the comparison between births with nearly complete exposure to those fully exposed to the tax increase from conception around the cut-off point.

## 4 Results

Table 2 shows the main results from the regression discontinuity analysis. Our model specifications vary by column as we integrate more controls. These mother-level controls include birth order (including first, second, and third or higher-order births), indicators for prenatal care reception, marital status, educational attainment, insurance coverage, and the mother’s occupation. Our results from incorporating variables related to maternal characteristics as covariates remain consistent and do not alter the conclusions. This alignment can be observed by comparing columns (1) and (2) with column (3) in Table 2. The fact that including covariates does not significantly alter point estimates suggests that these covariates are predetermined in our base specification (Calonico et al., 2019). Column 1 estimates include week-of-the-year and municipality fixed effects, column 2 includes the unemployment rate, and finally, in column 3 we include mother sociodemographic characteristics as controls. Our findings include robust bias-corrected effects and standard errors (Calonico et al., 2019).

The results of panel A show that there was an increase in the average infant birth weight of approximately 10 g, which represents a 0.3% increase with respect to the average birth weight before tax. This result is statistically significant at a confidence level of 95%. Our preferred specification includes all controls (column 3). To measure the effects of the tobacco tax along the birth weight distribution, we estimate its effect on the probability of having a low birth weight, normal birth weight, and fetal macrosomia. The results for these variables are not statistically significant, so we cannot draw any conclusions.

**Table 2:** Estimation results for birth weight outcomes.

	(1)	(2)	(3)
Panel A: Weight in grams			
RD Estimate	9.6362*	9.6650*	10.2745**
	(5.0286)	(5.0207)	(5.2309)
Mean (left)	3164.2037	3164.2037	3164.2037
Observations	822,244	822,244	822,244
Bias Corrected Effect	11.9092	11.9350	12.6030
Robust Std. Error	5.5300	5.5166	5.7620
Panel B: Low birth weight			
RD Estimate	−0.0036	−0.0036	−0.0036
	(0.0029)	(0.0029)	(0.0029)
Mean (left)	.0684	.0684	.0684
Observations	822,244	822,244	822,244
Bias Corrected Effect	−0.0047	−0.0048	−0.0047
Robust Std. Error	0.0033	0.0033	0.0033
Panel C: Normal birth weight			
RD Estimate	0.0018	0.0019	0.0016
	(0.0033)	(0.0033)	(0.0033)
Mean (left)	.8972	.8972	.8972
Observations	822,244	822,244	822,244
Bias Corrected Effect	0.0026	0.0027	0.0025
Robust Std. Error	0.0039	0.0039	0.0039
Panel D: Fetal macrosomia			
RD Estimate	0.0015	0.0015	0.0016
	(0.0020)	(0.0020)	(0.0020)
Mean (left)	.0345	.0345	.0345
Observations	822,244	822,244	822,244
Bias Corrected Effect	0.0021	0.0021	0.0023
Robust Std. Error	0.0023	0.0023	0.0023
Week & Mun. & Year FE	Yes	Yes	Yes
Unemployment rate	No	Yes	Yes
Sociodem. Charact.	No	No	Yes

Notes: This table estimates the effect of the tax on weight related outcomes including (a) birth weight in grams, (b) low birth weight, (c) normal birth weight and (d) fetal macrosomia using a RD methodology with time as the running variable and the Tax serving as the treatment dummy. Data used for this analysis are drawn from birth records spanning 2008 to 2019, with individual births as the unit of observation. Each column corresponds to a distinct regression using January 1st as the cutoff and assigning treatment based on exposure determined based on the date at entering the third trimester (3rd tri). Residuals of the outcome variables are used as dependent variables. Column (1) displays results after partialling out municipality, week of the year fixed effects, and year fixed effects. In column (2), the model additionally accounts for the unemployment rate, while column (3) incorporates sociodemographic characteristics as covariates. All estimations consist of a local linear regression of a second degree polynomial with triangular kernel weights and a 90-day bandwidth, estimated using rdrobust in Stata. * .10 ** .05 *** .01 sig. levels. Robust standard errors in parentheses.

To better understand whether these average effects mask important variation across subpopulations, we next examine heterogeneity in the impact of the tobacco tax. This examination considers maternal characteristics, including age, education, birth order, and marital status. The maternal age cohorts were divided into three age brackets: 15–19 years, 20–29 years, and 30–44 years. The educational level was classified into mothers without education, those with less than high school, high school graduates, and those with higher or collegiate qualifications. The results were also estimated for whether it was the mothers first live birth, the second birth, or a higher-order birth. Moreover, we did separate estimations for single mothers (never married, divorced, or widowed), and nonsingle mothers, which includes those presently married or in cohabitation. Our analysis of maternal demographic characteristics suggests that improvements in terms of birth weight were more pronounced among mothers with less than high school education, who were not single and aged 30 to 44 years. Among college-educated mothers, we observe slight decreases in normal birth weight, but no effects on low birth weight or fetal macrosomia. However, these findings are only significant at the 10 percent level. We find no meaningful differences in birth weight by birth order. Detailed results by maternal characteristics appear in Tables B1 through Table B4 in the Appendix.

In addition to our main outcomes, we also looked at how the tax affected other health indicators. These include respiratory complications, gestational duration, extreme cases of low birth weight, neonatal length, and congenital anomalies. Figures A5 and A6 show the main results for the supplementary variables using de-meaned and seasonally adjusted residuals. The patterns in the figures are consistent with the point estimates from the statistical models.

Existing studies show that maternal smoking during pregnancy impedes fetal lung development, leading to weakened respiratory mechanics and reduced lung function (Spindel & McEvoy, 2016). To evaluate effects on infant respiratory health, we used several metrics based on the Silverman–Anderson score. The score, which ranges from 0 to 10, measures newborn respiratory distress at birth. We used four indicators: the raw Silverman score; any respiratory difficulty (score ≥1); moderate to severe issues (score ≥4); and severe distress (score ≥7). Table A1 reports the results for the Silverman score and the derived indices. We find no significant effect on the Silverman score or on the likelihood of respiratory problems.

Previous research indicates that maternal smoking during pregnancy is also linked to premature birth and reduced gestational age (Wen et al., 1990). To examine effects on gestational age, we use two measures: gestational age in weeks and an indicator for preterm births (less than 37 weeks). The results show no significant changes in the average gestational age or in the prevalence of preterm births. The corresponding results are shown in Table A2. Postterm births occurred in only 0.01% of the cases, making it difficult to assess any effect related to the tax policy.

When examining extreme outcomes concerning birth weight, impacts are overall insignificant. We find a small but statistically significant increase in newborn length of $0.06 centimeters, yet its practical impact for policy purposes is minimal.

Previous studies have linked smoking during pregnancy to several types of congenital anomalies (Hackshaw et al., 2011). To examine these outcomes, our analysis includes anomalies in the cardiovascular and cardiac systems, irregularities in the musculoskeletal structure, deviations in facial features, variations in the ocular structure, instances of orofacial clefts, irregularities in the gastrointestinal system, hernias, hypospadias, and imperfections in the skin. Across these measures, we find no statistically significant changes following the tax increase. These conditions are also very rare—just 0.05% of the sample—limiting our ability to detect statistically meaningful effects.

### 4.1 Robustness checks

**Fig. 2:**
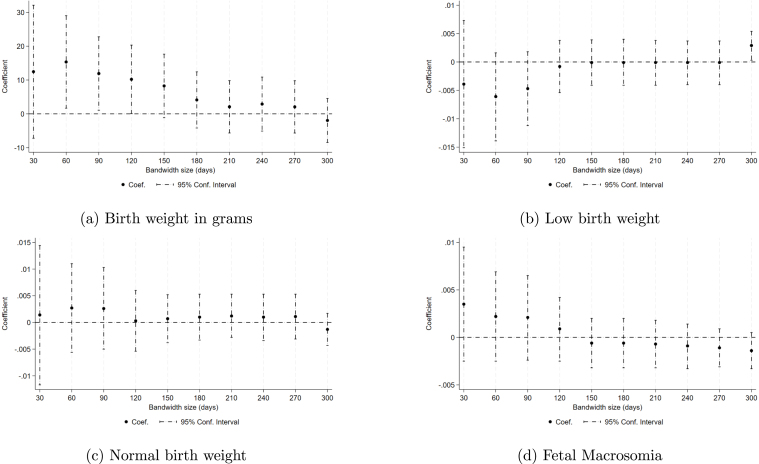
Tax effect on birth weight by bandwidth. *Notes:* This figure displays point estimates for the effect of the tax on (a) birth weight in grams, (b) low birth weight, (c) normal birth weight and (d) fetal macrosomia using a RD methodology with time as the running variable and the Tax serving as the treatment dummy. Each data point originates from a distinct regression using January 1st as the cutoff and assigning treatment based on exposure determined based on the date at entering the third trimester (3rd tri) but uses a different bandwidth of days before and after the tax rate change. Data used for this analysis are drawn from birth records spanning 2008 to 2019, with individual births as the unit of observation. Each regression included is based on a specification using de-meaned and seasonally adjusted residuals and includes sociodemographic characteristics as controls.

This section presents additional robustness checks to test the reliability of our main findings. We re-estimate the tax effects using varying time windows centered around the cut-off, ranging from 30 days to 300 days. Our preferred choice is a three-month window, the length of one trimester. Although wider time windows improve statistical certainty, they also increase the risk of introducing spurious variation due to potential temporal trends and unmeasured factors. Our results, including observations farther away from the date of the tax hike, tend to converge toward zero, while results closer to the implementation date suggest potential improvements, however, most estimates are insignificant, as can be observed in Fig. 2 . We see similar patterns for low birth weight, gestational length in weeks, preterm pregnancies, and height, as shown in A1 and A2, although all estimates in these cases are not significant.

We also conduct placebo tests through parallel regression discontinuity analyses using alternative dates from 2008 to 2019 with January 1st as the cut-off point. This method tests if our results are not just a “new year” effect, meaning an effect that comes from a change in the calendar year and the seasonality associated with the annual adjustment for inflation in the Special Tax on Production and Services (IEPS) rate, implemented on the first day of each year. Fig. 4 shows the results of the independent analysis performed for all dates of January 1 between 2009 and 2019. The results show that 2011 is the deviation from the trend in all outcomes. We also performed tests that compared behaviors before and after the announcement date using November 19, 2010 as the cutoff date. The analysis did not result in significant behavior changes. The results of this analysis on our main variables of interest are shown in Figure C1.

**Fig. 3:**
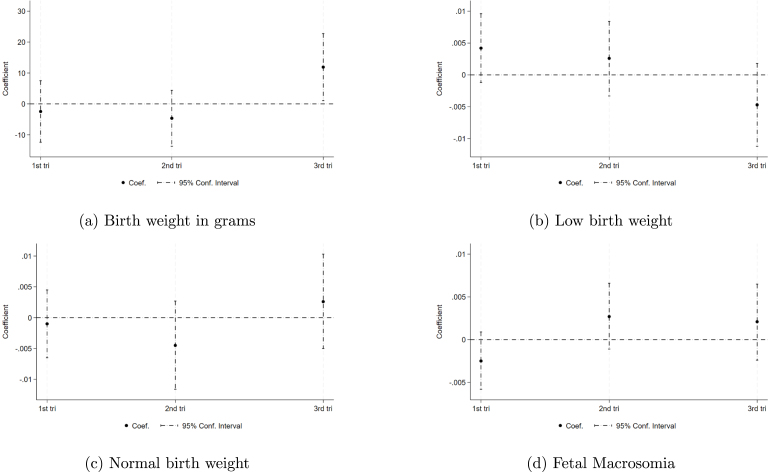
Tax effect on Birth Weight by Trimester of Gestation. *Notes:* This figure displays point estimates for the effect of the tax on (a) birth weight in grams, (b) low birth weight, (c) normal birth weight and (d) fetal macrosomia using a RD methodology with time as the running variable and the Tax serving as the treatment dummy. Each data point originates from a distinct regression using January 1st as the cutoff and assigning treatment based on exposure determined at the date entering the first trimester (1st tri), the date at entering second trimester (2nd tri), and the date at entering third trimester (3rd tri). Each data point uses a bandwidth of 90 days before and after the tax rate change. Data used for this analysis are drawn from birth records spanning 2008 to 2019, with individual births as the unit of observation. Each regression included is based on a specification using de-meaned and seasonally adjusted residuals and includes sociodemographic characteristics as controls.

We also examined how timing of exposure during pregnancy matters by categorizing women into the intervention group based on their entry into the first, second, and third trimesters. The first trimester is defined as encompassing weeks 0–13, the second trimester spans from week 13 to week 27, and the third trimester extends from week 28 on. The weeks of gestation are directly obtained from the birth records data. Positive impacts in birth weight are observed when exposure is determined by entering the third trimester. No significant impacts are observed for low birth weight, normal birth weight, or fetal macrosomia. These results are depicted in Fig. 3. This finding aligns with similar studies and evidence suggesting that the third trimester of gestation is a critical period marked by rapid fetal growth and lung development.

We also explore models that include control variables related to maternal characteristics, without imposing specific parametric constraints. These mother-level controls encompass various birth record variables, such as birth order (including first, second, and third or higher-order births), indicators for prenatal care reception, marital status, educational attainment, insurance coverage, and the mother’s occupation. Our results from incorporating variables related to maternal characteristics as covariates remain consistent and do not alter the conclusions. This can be seen by comparing columns (1) and (2) with column (3) in Table 2. The fact that including covariates does not significantly alter point estimates suggests that these covariates are predetermined in our base specification (Calonico et al., 2019).

We additionally explored the sensitivity of our results to the exclusion of observations with extreme birth weights, specifically those below 500 g or above 7000 g. When we include these extreme values of birth weight, the estimated birth weight gain drops slightly to 7 g and is no longer statistically significant. However, estimates using different bandwidths centered on the policy implementation date produce similar patterns to our main analysis. This suggests that excluding potential data entry errors improves statistical precision, but does not drive the direction of the effect. These results are illustrated in Figure C2 and Figure C3 in the Appendix.

As a placebo test, we check whether the tax affected unrelated outcomes to demonstrate continuity as proposed by Hausman and Rapson (2018). The analysis suggests the impacts are either statistically insignificant or indicate precise null effects as shown in Figure A3 in the Appendix. Similarly, Figure C4 in the Appendix also confirms that these factors do not exhibit discontinuity at the cut-off.

**Fig. 4:**
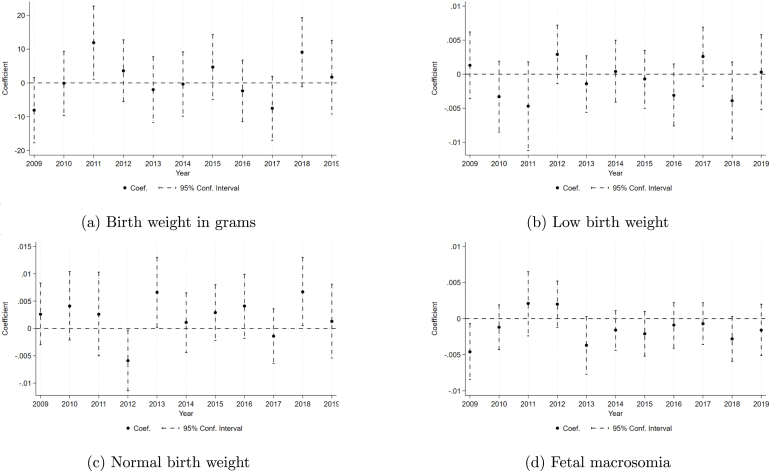
Tax effect of alternate January 1st placebo dates on birth weight. *Notes:* This figure displays point estimates for the effect of the tax on (a) birth weight in grams, (b) low birth weight, (c) normal birth weight and (d) fetal macrosomia using a RD methodology with time as the running variable and the Tax serving as the treatment dummy. Each data point comes from a different regression using January 1st of that year as the cutoff assigning treatment based on exposure determined based on the date at entering the first third (3rd tri). Each data point uses a bandwidth of 90 days before and after the tax rate change. Data used for this analysis are drawn from birth records spanning 2008 to 2019, with individual births as the unit of observation. Each regression included is based on a specification using de-meaned and seasonally adjusted residuals and includes sociodemographic characteristics as controls.

To address immediate selection concerns, we conducted our analysis excluding intervals centered alternatively on 15, 30, 45, and 60 days around January 1, 2011. Our findings, illustrated in Figure C5 in the Appendix, suggest evidence indicating the impacts of the policy may be negligible when focusing on data further away from the implementation date. These findings align with our results of estimating effects using different bandwidths. This may indicate that responses to the policy are only immediate or that the period around the policy implementation may coincide with other public health interventions or external events.

Finally, we assessed the impact of the tax increase outside of the discontinuity to obtain additional insights. This is inherently challenging as considering observations too far off the implementation of the policy can lead to confounded effects. Our approach consists of differentiating treatment based on the number of trimesters of exposure for women who were already pregnant at the time of the tax introduction. Details about the methodology are provided in section D of the Appendix. Our analysis found that one trimester of exposure to the tax led to a 5–6 gram increase in birth weight, with no improvements observed for three trimesters. However, longer exposure (three trimesters) was associated with reductions in low birth weight and increases in macrosomia, along with a shift in the weight distribution toward higher weights. All comparisons use two trimesters of exposure as the reference category. These findings align with the regression discontinuity analysis for weight, while providing additional insight on low birth weight. The results are presented in Table D1 of the Appendix.

## 5 Discussion

### 5.1 Study findings

Our findings suggest that the tobacco tax increase—equivalent to a 36 percent rise in cigarette prices (Reynales-Shigematsu et al., 2020), resulted in a birth weight gain of up to 10 g. Our findings are consistent with previous evidence from the United States: Lien and Evans (2005), for example, reported increases in birth weight of 8 g in Arizona and 11 g in Michigan following a 30 percent cigarette price hike. We find, however, no significant changes in the prevalence of low birth weight or fetal macrosomia. This distinction is important: improvements in average birth weight do not necessarily translate into shifts at the extremes of the distribution, where risks to newborn and later-life health are most concentrated (Bianchi and Restrepo, 2022, Risnes et al., 2011). Low birth weight is, for example, linked to higher mortality in adulthood due to cardiovascular causes (Risnes et al., 2011). However, this relationship is U-shaped, which means that only very low and very high birth weights are associated with elevated mortality risks (Risnes et al., 2011). Fetal macrosomia, carries its own set of complications, including later-life obesity (Zhao et al., 2012). Similarly, prior research links low birth weight, not solely decreases in birth weight, to poorer adult health and lower educational attainment (Royer, 2009). Since we did not find a significant impact on the extremes of the birth weight distribution, we caution that our findings cannot be interpreted as indicative of individual or long-term benefits beyond the modest gains in fetal growth observed.

The observed improvements in birth outcomes were especially notable among children of mothers with less than a high school education. This pattern aligns with previous evidence from the United States, where tobacco tax increases have been shown to benefit socioeconomically disadvantaged mothers. For example, Hawkins et al. (2014) found that higher taxes on cigarettes were associated with gains in birth weight of 5 g among White mothers and 4 g among Black mothers with low educational attainment. Similarly, Hawkins and Baum (2019) reported that a $1 tax increase was associated with increases of 4.2 and 0.9 g, respectively, among infants born to white and Black mothers with low education. A more recent study by Hawkins et al. (2025) found that a tax increase of $1.00 increased birth weight by 7.7 g for women under 12 years of education. The effects for more educated mothers were smaller but still statistically significant.

We also find stronger effects among mothers who were married or living with a partner. Marital or partnership status is not a commonly disaggregated dimension in most studies evaluating the impact of tobacco taxation on birth outcomes. However, some evidence supports marriage protection, with prenatal smoking mediating the relationship between marital status and birth weight (Kane, 2016). This pattern may reflect contextual differences in the way the household context shapes tobacco exposure or cessation support.

Our findings also indicate that the largest effects were among mothers aged 30–44 years. This pattern contrasts with evidence from the United States, where modest positive effects of tobacco taxes on birth weight have been observed mainly among teenage mothers (Markowitz et al., 2011). This difference may reflect variation in demographics or health behaviors between countries. For example, older mothers in Mexico may respond more to public health messages than younger mothers. These contextual variations suggest that maternal age may interact with policy effectiveness in different ways across countries-an area worth exploring further.

We find no clear evidence that the tax produced broader improvements in newborn health beyond birth weight. Specifically, we examined outcomes such as respiratory complications, gestational age, height at birth, and congenital anomalies, conditions that are also known to be affected by prenatal tobacco exposure. Bandwidth-specific analyses, however, suggest small marginal benefits near the policy cutoff for very low birth weight, extreme low birth weight, gestational length and preterm status following the tax increase. Although these findings are not statistically significant, the estimates are precise due to the use of the full population of birth records.

While the individual-level gains in birth weight found are modest, their potential implications for population health should not be overlooked. From a clinical perspective, a 10-gram increase in birth weight is generally not considered meaningful on an individual level. Clinical thresholds for concern or intervention typically focus on more substantial differences—such as moving a newborn out of the low birth weight category or preventing outcomes associated with very low or macrosomic birth weights. However, the fact that the largest gains we observe are among mothers with lower education and in older age brackets suggests that taxation may be especially effective among socioeconomically vulnerable populations. Hawkins et al. (2025) support this notion by demonstrating that increasing state-level cigarette taxes contributes to diminishing educational inequalities in prenatal smoking. Moreover, even small individual improvements, when aggregated at the population level, could yield significant long-term public health benefits.

### 5.2 Limitations

While our design and use of national birth registry data offer clear strengths, the study also has important limitations. A key limitation is that there is no cross-sectional variation in treatment status. The absence of a natural control group due to the tax being implemented at the national level poses challenges in determining comparison groups to assess long-term developmental outcomes of the policy. This makes it difficult to separate the effects of the tax from other concurrent tobacco control policies, particularly over longer periods. Unlike some studies on national-level interventions that incorporate additional differences, this tax policy was uniformly applied without variations between different subjects or locations. Consequently, it was not feasible to employ a difference-in-discontinuities design.

Our main specifications focus on a short 90-day window before and after the tax increase. This approach helps minimize potential contamination from other policy changes implemented before the tax hike, including prior tax increases. Our primary concern with expanding the analysis window further is the introduction of pictorial warnings on cigarette packages, which became mandatory on September 24, 2010 and remained in place during the period we analyze. These warnings, which feature graphic health images, may have influenced smoking behavior. This policy could have affected prenatal smoking, potentially confounding the study findings. If the pictorial warnings had a positive effect, expanding the analysis window would make it more difficult to detect a positive impact attributable to the tax increase.

We restricted our analysis to data from 2008 to 2019 to focus on the pre-pandemic period. This allows us to use the longest reliable time series to model and remove seasonal patterns and long-run trends without risking contamination from pandemic-related disruptions (Hausman & Rapson, 2018). Although birth registration continued during the pandemic, mobility restrictions and staffing shortages in Mexico reduced the flow of registry data and may have introduced errors in the recorded birth dates.

One of the main limitations, as mentioned throughout the document, is the lack of consistent annual data on tobacco or cigarette consumption in Mexico. Although it may be tempting to explore heterogeneity in policy effects using a pre-post interaction with state-level smoking prevalence, we do not pursue this approach due to data constraints. The most recent available estimates of smoking rates by state precede the policy by three years (2008), limiting their usefulness in capturing baseline conditions at the time of the 2011 reform. A pre-post analysis of this kind would require assuming minimal variation in smoking trends across states between 2008 and 2011, an assumption that is unlikely to hold given the concurrent tobacco measures that occurred during this period.

Another limitation in our study is the absence of information in birth records on the smoking behavior of the expectant mother and others living in the same household, including partners. Although we cannot directly assess changes in maternal smoking, prior empirical estimates of cigarette price elasticity provide indirect support for our findings (Jiménez-Ruiz et al., 2008, Olivera-Chávez et al., 2010). Lack of this information, however, impedes our ability to differentiate between the impacts on mothers who smoke and those who do not. Given that we cannot separately identify the effects of reduced active smoking and reduced second-hand smoke exposure, our findings should be interpreted as capturing the combined impact of both pathways.

Finally, another limitation is we are unable to distinguish the specific effects of the tax increase on women who knew about their pregnancy from the outset versus those who discovered it later during gestation. In Mexico, 98.6% of women are aware of the health risks associated with smoking (CONADIC, 2017). If women who are aware of their pregnancy actively start quitting due to the tax, we might expect varying impacts depending on the timing of their pregnancy awareness.

### 5.3 Directions for future research and policy implications

Despite growing evidence on the health benefits of tobacco control, critical gaps remain—especially in low- and middle-income countries—regarding the specific impacts of taxation on maternal and newborn health. Our study adds new evidence from a middle-income country context. As mentioned in Section 5.1, the magnitude of our estimates is comparable to findings from studies in the United States that specifically assess the impact of tobacco taxation on birth outcomes (Hawkins and Baum, 2019, Hawkins et al., 2014, Hawkins et al., 2025, Lien and Evans, 2005). Existing research outside of the United States tends to assess tax policies alongside other tobacco control measures—such as smoke-free laws, media campaigns, or warning labels—or focuses exclusively on non-tax interventions. For example, Peelen et al. (2016) find that smoke-free legislation in workplaces, coupled with a tobacco tax increase, and a media campaign, decreased the odds of being small for gestational age in the Netherlands. The gap in evidence related to tax policy and birth outcomes is reflected in global reviews. The systematic review by Akter et al. (2023) found that smoke-free legislation is associated with improved birth outcomes. However, no associations of tobacco tax policies with health-related outcomes in general, including birth outcomes, were found. This is primarily due to the limited number of available studies.

A broader regional outlook is particularly required in Latin America, where constraints in data and infrastructure continue to be a challenge. The limited comparisons available within the region reveal considerable variation in policy effectiveness. In Uruguay, tax increases had limited efficacy in reducing tobacco smoking, allegedly due to industry price responses and lobbying activity, and, in contrast, national non-price policies had the estimated largest impact on cessation (Barnoya and Glantz, 2002, Harris et al., 2015). In Peru, a combination of non-tax policies, including smoke-free laws, graphic warnings, and restrictions on tobacco sales to minors, were linked to reductions in premature births, although the effects on birth weight were negligible (Mallma et al., 2020). Specifically, Mallma et al. (2020) found birth weight gains of no more than 0.85 g, suggesting that stronger or more comprehensive strategies may be needed to improve maternal and newborn health. While our main findings point to moderate short-term gains, future research should explore longer-term developmental effects of prenatal exposure to tobacco taxation and compare Mexico’s experience with that of other low- and middle-income countries that undergo similar fiscal reforms. Cross-national analyses of differences in price responsiveness, policy enforcement, and industry behavior could offer important information to maximize taxation health returns.

The cost associated with smoking accounts for approximately 0.4% of the GDP in Mexico. Tax revenues obtained from tobacco sales in Mexico recover 45% of the health expenditures attributed to smoking (Pichon-Riviere et al., 2016). Tobacco taxes in Mexico remain lower than in many high-income countries, suggesting room for further increases (Huesca et al., 2021). Although taxation is among the most effective tobacco control tools, it is often implemented alongside other regulatory measures, such as advertising restrictions, public smoking bans, or packaging requirements, complicating efforts to isolate its specific impact. Strategic policy design can help a clearer understanding of the relative contribution of each component and could allow future work to examine how fiscal measures interact with other complementary policies aimed at reducing prenatal tobacco exposure, such as targeted smoking cessation programs for pregnant women, public education campaigns, or smoke-free housing initiatives. However, the design and implementation of tobacco tax policy must be tailored to national circumstances to ensure effectiveness.

Improving the data infrastructure is also essential to generate policy-relevant evidence. We recommend that future data systems in Mexico and similar contexts integrate standardized indicators of maternal smoking and exposure to household tobacco into birth registries. In addition, high-frequency surveillance systems should be developed to track tobacco use, including both active smoking and second-hand exposure. Such data are critical to distinguish the effects of direct versus indirect exposure and to assess variation in policy impact in household contexts. Stronger measurement capacity would improve the ability of researchers and policy makers to identify who benefits the most under different tobacco control strategies.

## 6 Conclusions

This study evaluated the effects of a major tobacco tax increase in Mexico in 2011 on birth weight and related outcomes. We use a distinctive methodology, a time-based regression discontinuity analysis, to address the challenges posed by the uniform implementation of a nationwide reform. The results suggest that large tobacco tax hikes can lead to modest but measurable short-term improvements in birth outcomes in middle-income settings, particularly in birth weight. Our findings underscore the need to strengthen fiscal policy tools for maternal and child health and to improve the data infrastructure needed to track behavioral responses and health impacts over time.

## CRediT authorship contribution statement

**Francisco Beltran-Silva:** Writing – review & editing, Writing – original draft, Visualization, Project administration, Methodology, Investigation, Formal analysis, Data curation, Conceptualization. **Rodrigo Aranda:** Writing – review & editing, Visualization, Validation, Methodology, Investigation, Formal analysis, Conceptualization.

## Ethical statement

This study uses publicly available, anonymized birth records from Mexico’s national vital statistics system, maintained by the Ministry of Health. The data contain no personal identifiers, and no human subjects were directly involved. In accordance with institutional guidelines, research based solely on de-identified public data does not require approval from an ethics review board. Therefore, ethical approval was not sought.

## Funding

I declare that no funding was received for this research project, and as a result, there was no financial support that could have influenced the outcome.

## Declaration of competing interest

The authors declare that they have no known competing financial interests or personal relationships that could have appeared to influence the work reported in this paper.

## Data Availability

Data will be made available on request.

## Appendix A Supplementary data

MMC S1 Supplementary material with robustness checks, heterogeneity analysis, and additional newborn outcomes.

## Footnotes

1 Global Burden of Disease [database]. Washington, DC: Institute of Health Metrics; 2019. IHME.

2 While excluding extreme birth weights is common in research using administrative data, few published articles explicitly justify these specific thresholds in their methodological sections. We follow Ludwig and Currie (2010) in setting these limits, which helps minimize the influence of implausible or miscoded values and improves internal validity. We also estimated our preferred specification without excluding these extreme cases. The results are presented in Appendix Figures C2 and C3.
