# Supplementary material for: Taxing for healthier beginnings: The impact of a major tobacco tax hike on birth weight in Mexico
Source: SSM Popul Health. 2025 Aug 26;32:101851. doi: 10.1016/j.ssmph.2025.101851 (PMC12744650; doi:10.1016/j.ssmph.2025.101851)
Supplement: MMC S1 — Supplementary material with robustness checks, heterogeneity analysis, and additional newborn outcomes. [file mmc1.pdf]

9 Appendix

A Results for Supplementary Outcomes

Table A1: Estimation Results for Respiratory Problem Outcomes

	(1)	(2)	(3)
Panel A: Silverman score			
RD Estimate	-0.0010	-0.0007	0.0007
	(0.0106)	(0.0106)	(0.0107)
Mean (left)	.2109	.2109	.2109
Observations	810,477	810,477	810,477
Bias Corrected Effect	0.0015	0.0019	0.0034
Robust Std. Error	0.0125	0.0125	0.0126
Panel B: Any respiratory problems			
RD Estimate	-0.0011	-0.0011	-0.0007
	(0.0025)	(0.0025)	(0.0026)
Mean (left)	.1048	.1048	.1048
Observations	810,477	810,477	810,477
Bias Corrected Effect	-0.0009	-0.0009	-0.0005
Robust Std. Error	0.0031	0.0031	0.0031
Panel C: Mild to severe respiratory problems			
RD Estimate	-0.0005	-0.0004	-0.0003
	(0.0012)	(0.0012)	(0.0012)
Mean (left)	.0099	.0099	.0099
Observations	810,477	810,477	810,477
Bias Corrected Effect	-0.0004	-0.0004	-0.0003
Robust Std. Error	0.0014	0.0014	0.0014
Panel D: Severe respiratory problems			
RD Estimate	0.0003	0.0003	0.0004
	(0.0010)	(0.0010)	(0.0010)
Mean (left)	.0061	.0061	.0061
Observations	810,477	810,477	810,477
Bias Corrected Effect	0.0006	0.0006	0.0008
Robust Std. Error	0.0012	0.0012	0.0012
Week & Mun. & Year FE	Yes	Yes	Yes
Unemployment rate	No	Yes	Yes
Sociodem. Charact.	No	No	Yes

Notes: This table estimates the effect of the tax on outcomes related to respiratory problems including (A) Silverman score, (B) any respiratory problems, (C) Mild to severe respiratory problems and (D) Severe respiratory problems using a RD methodology with time as the running variable and the Tax serving as the treatment dummy. Data used for this analysis are drawn from birth records spanning 2008 to 2019, with individual births as the unit of observation. Each column corresponds to a distinct regression using January 1st as the cutoff and assigning treatment based on exposure determined based on the date at entering the third trimester (3rd tri). Residuals of the outcome variables are used as dependent variables. Column (1) displays results after partialling out municipality, week of the year fixed effects, and year fixed effects. In column (2), the model additionally accounts for the unemployment rate, while column (3) incorporates sociodemographic characteristics as covariates. All estimations consist of a local linear regression of a second degree polynomial with triangular kernel weights and a 90-day bandwidth, estimated using rdrobust in Stata. * .10 ** .05 *** .01 sig. levels. Robust standard errors in parentheses.

Table A2: Estimation Results for Gestational Length Outcomes

	(1)	(2)	(3)
Panel A: Weeks of gestation			
RD Estimate	0.0307	0.0309	0.0213
	(0.0207)	(0.0207)	(0.0203)
Mean (left)	38.9148	38.9148	38.9148
Observations	822,244	822,244	822,244
Bias Corrected Effect	0.0393	0.0394	0.0292
Robust Std. Error	0.0235	0.0235	0.0230
Panel B: Pre-term birth			
RD Estimate	-0.0020	-0.0020	-0.0016
	(0.0027)	(0.0027)	(0.0027)
Mean (left)	.0518	.0518	.0518
Observations	822,244	822,244	822,244
Bias Corrected Effect	-0.0026	-0.0027	-0.0022
Robust Std. Error	0.0032	0.0032	0.0032
Week & Mun. & Year FE	Yes	Yes	Yes
Unemployment rate	No	Yes	Yes
Sociodem. Charact.	No	No	Yes

Notes: This table estimates the effect of the tax on gestational length outcomes including (A) gestational age in weeks and (B) pre-term pregnancy using a RD methodology with time as the running variable and the Tax serving as the treatment dummy. Data used for this analysis are drawn from birth records spanning 2008 to 2019, with individual births as the unit of observation. Each column corresponds to a distinct regression using January 1st as the cutoff and assigning treatment based on exposure determined based on the date at entering the third trimester (3rd tri). Residuals of the outcome variables are used as dependent variables. Column (1) displays results after partialling out municipality, week of the year fixed effects, and year fixed effects. In column (2), the model additionally accounts for the unemployment rate, while column (3) incorporates sociodemographic characteristics as covariates. All estimations consist of a local linear regression of a second degree polynomial with triangular kernel weights and a 90-day bandwidth, estimated using rdrobust in Stata. * .10 ** .05 *** .01 sig. levels. Robust standard errors in parentheses.

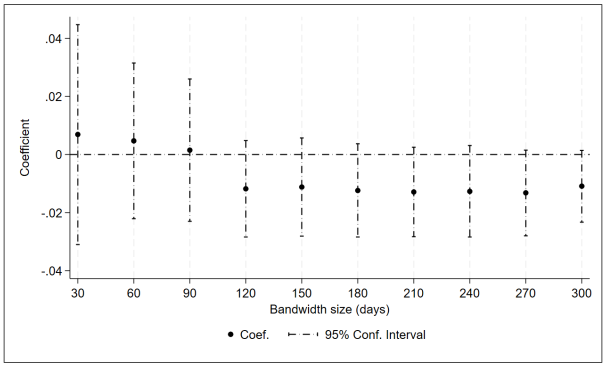

(a) Silverman score

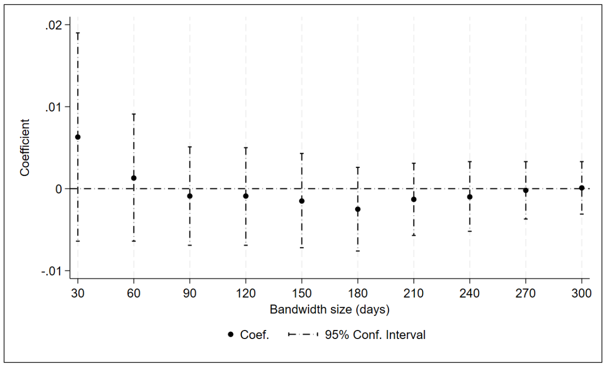

(b) Any respiratory problems

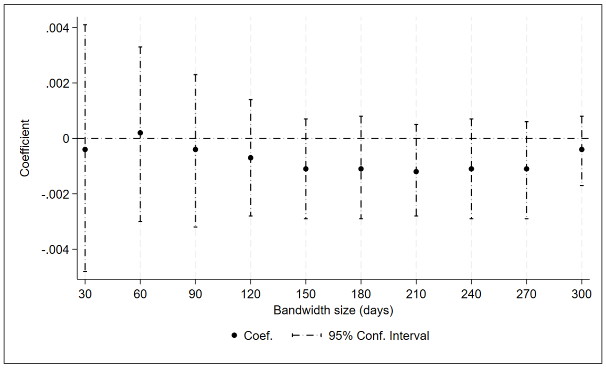

(c) Mild to severe respiratory problems

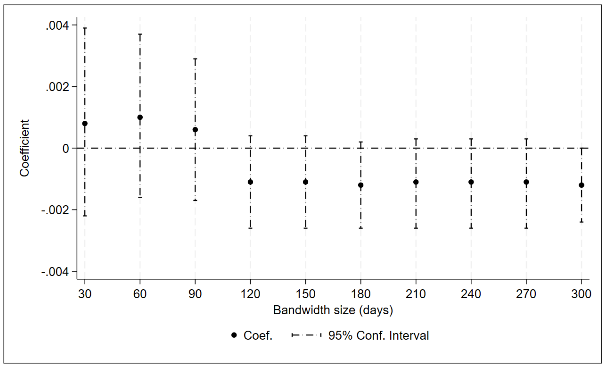

(d) Severe respiratory problems

Figure A1: Tax effect on respiratory problems by bandwidth. *Notes:* This figure displays point estimates for the effect of the tax on (a) Silverman Score, (b) Any respiratory problems, (c) Mild to severe respiratory problems and (d) Severe respiratory problems using a RD methodology with time as the running variable and the Tax serving as the treatment dummy. Each data point originates from a distinct regression using January 1st as the cutoff and assigning treatment based on exposure determined based on the date at entering the third trimester (3rd tri) but uses a different bandwidth of days before and after the tax rate change. Data used for this analysis are drawn from birth records spanning 2008 to 2019, with individual births as the unit of observation. Each regression included is based on a specification using de-meaned and seasonally adjusted residuals and includes sociodemographic characteristics as controls.

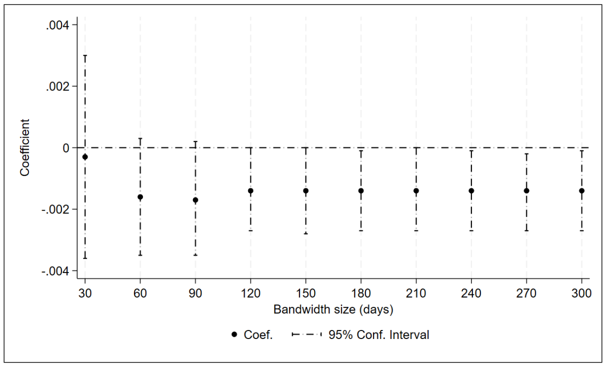

(a) Low birth weight (very)

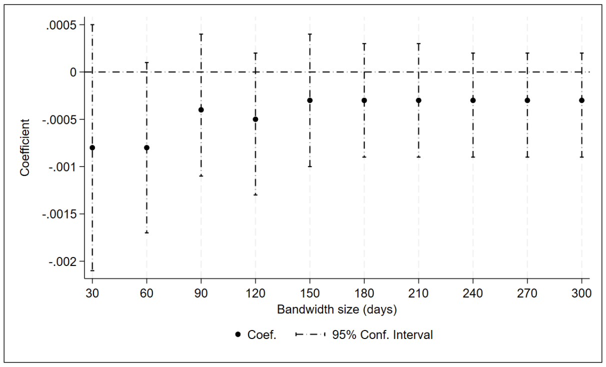

(b) Low birth weight (extreme)

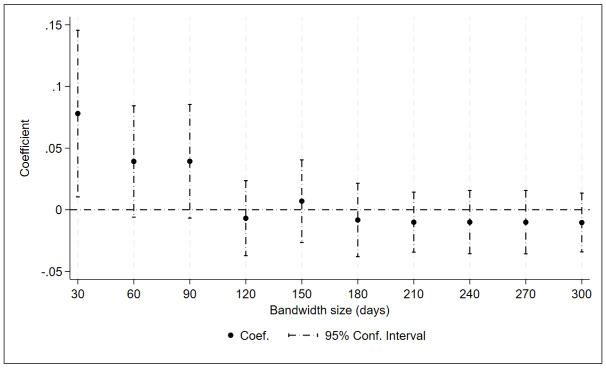

(c) Gestational length

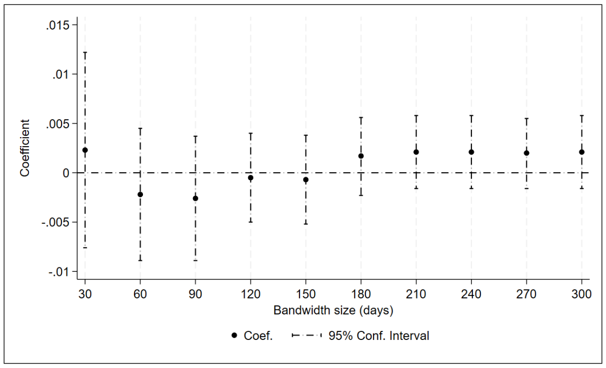

(d) Pre-term pregnancy

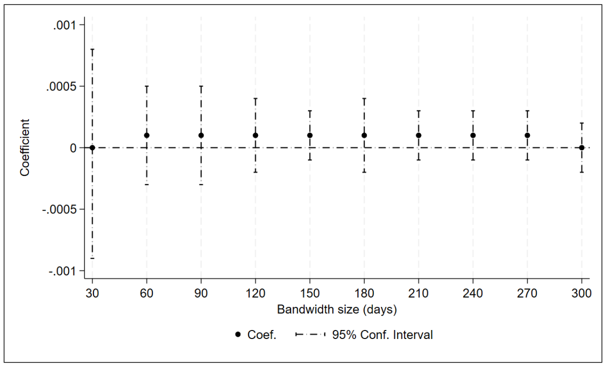

(e) Post-term pregnancy

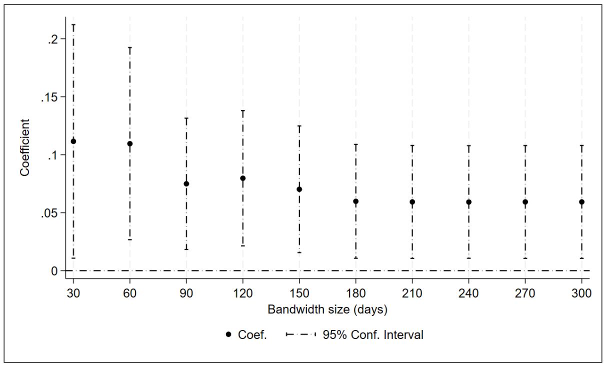

(f) Height

Figure A2: Tax effect on various outcomes related to gestational age, low birth weight and height by bandwidth. *Notes:* This figure displays point estimates for the effect of the tax on (a) Low birth weight (very), (b) Low birth weight (extreme), (c) gestational length, (d) pre-term pregnancy, (e) post-term pregnancy and (f) height using a RD methodology with time as the running variable and the Tax serving as the treatment dummy. Each data point originates from a distinct regression using January 1st as the cutoff and assigning treatment based on exposure determined based on the date at entering the third trimester (3rd tri) but uses a different bandwidth of days before and after the tax rate change. Data used for this analysis are drawn from birth records spanning 2008 to 2019, with individual births as the unit of observation. Each regression included is based on a specification using de-meaned and seasonally adjusted residuals and includes sociodemographic characteristics as controls.

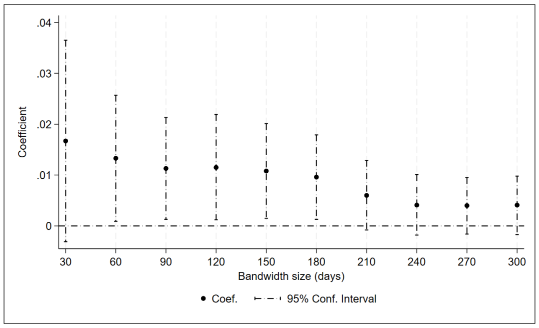

(a) Birth sex being female

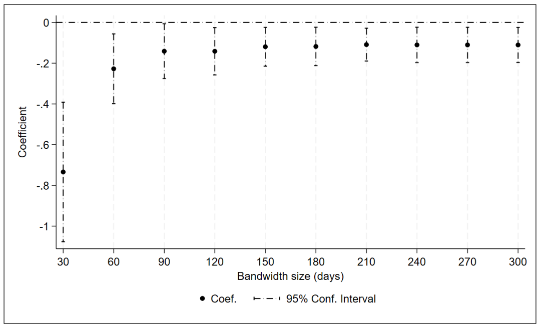

(b) Age of the mother

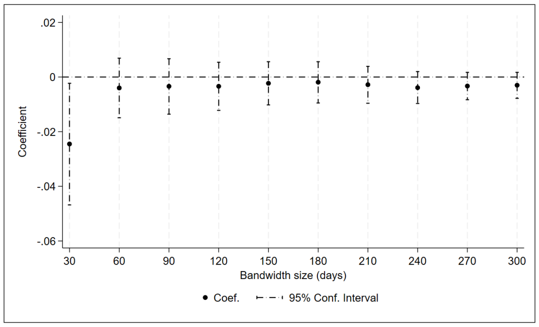

(c) Marriage status

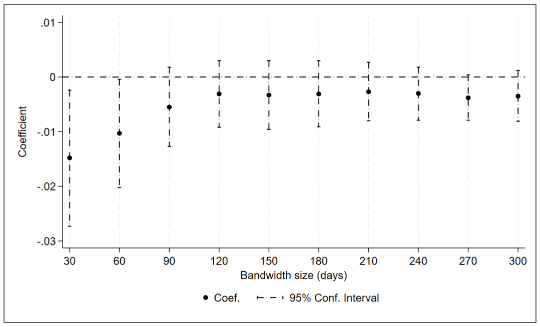

(d) Working Status

Figure A3: Effects on unrelated or placebo outcomes. Notes: The graph displays displays point estimates for the effect of the tax on (a) Birth sex being female, (b) age of the mother, (c) marriage status, (d) working status. To estimate the impact of the tax rate increase on January 1st, 2011, residuals are used to generate a scatter plot. The date at entering the third trimester relative to the cut-off date is used as the exposure of interest. Data used for this analysis are drawn from birth records spanning 2008 to 2019, with individual births as the unit of observation.

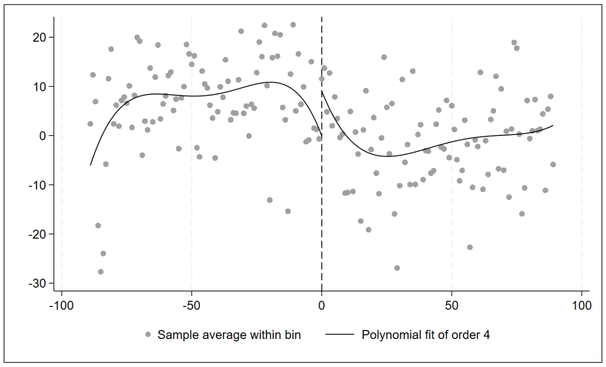

(a) Birth weight in grams

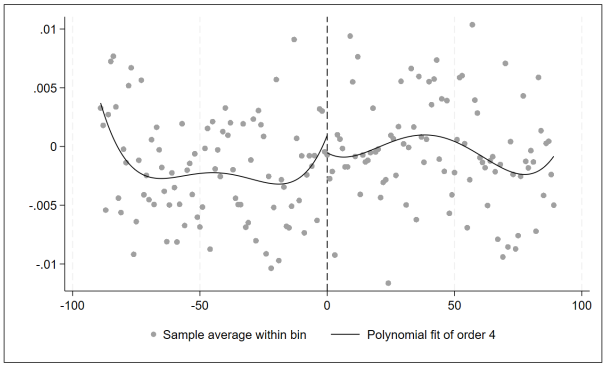

(b) Low birth weight

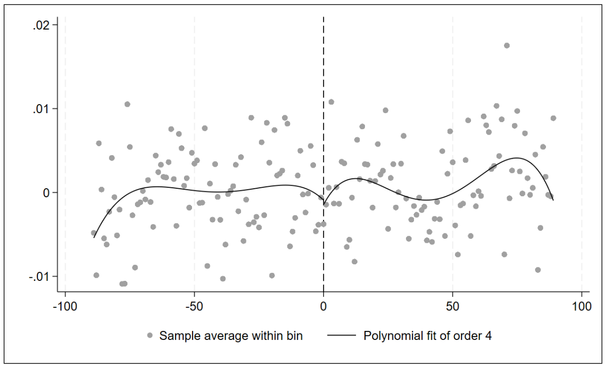

(c) Normal birth weight

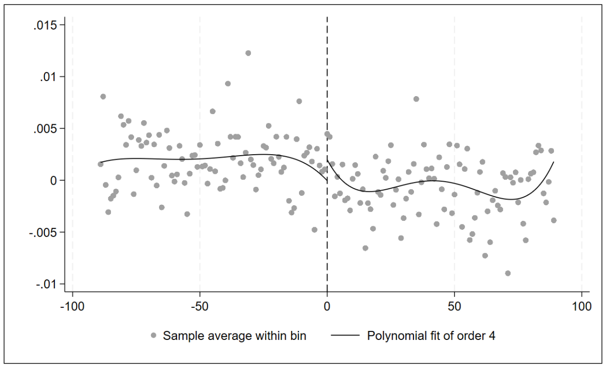

(d) Fetal macrosomia

Figure A4: Tax effect on birth weight, graphical analysis. *Notes*: The graph displays the de-meaned and seasonally adjusted residuals for various outcomes related to birth weight: (a) birth weight in grams, (b) low birth weight, (c) normal birth weight and (d) fetal macrosomia. To estimate the impact of the tax rate increase on January 1st, 2011, residuals are used to generate a scatter plot. The date at entering the third trimester relative to the cut-off date is used as the exposure of interest. This plot is then utilized to make separate polynomial predictions for both 90 days before and after the tax rate change while controlling for sociodemographic characteristics. The vertical line refers to January 1st 2011. Data used for this analysis are drawn from birth records spanning 2008 to 2019, with individual births as the unit of observation.

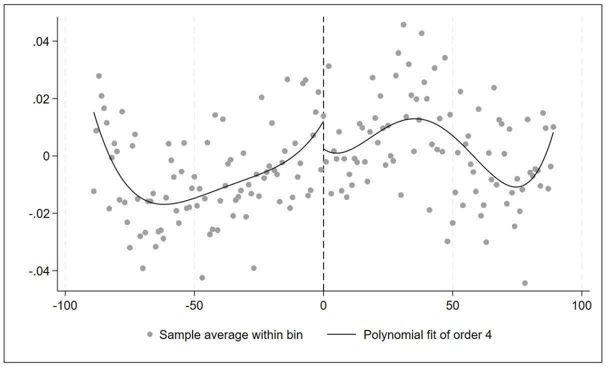

(a) Silverman score

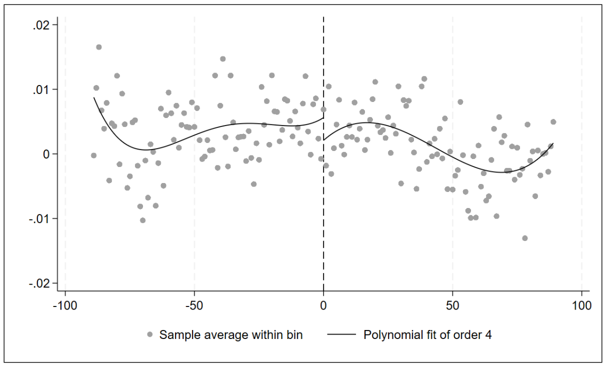

(b) Any respiratory problems

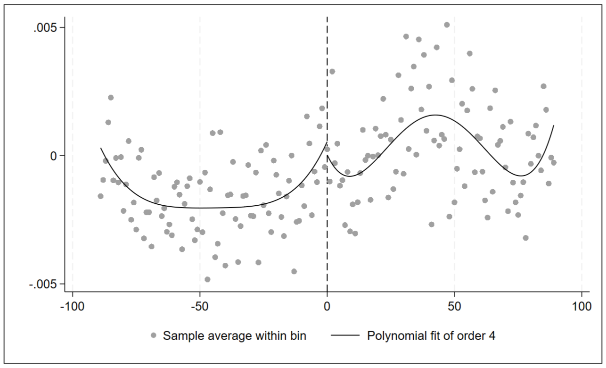

(c) Mild to severe respiratory problems

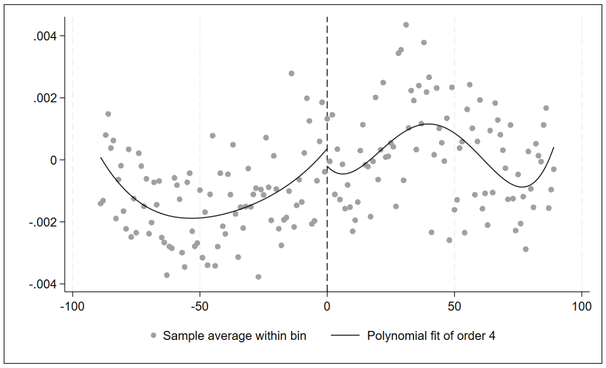

(d) Severe respiratory problems

Figure A5: Tax effect on respiratory problems. *Notes*: The graph displays the de-meaned and seasonally adjusted residuals for various outcomes related to respiratory problems: (a) Silverman Score, (b) Any respiratory problems, (c) Mild to severe respiratory problems and (d) Severe respiratory problems. To estimate the impact of the tax rate increase on January 1st, 2011, residuals are used to generate a scatter plot. The date at entering the third trimester relative to the cut-off date is used as the exposure of interest. This plot is then utilized to make separate polynomial predictions for both 90 days before and after the tax rate change while controlling for sociodemographic characteristics. The vertical line refers to January 1st 2011. Data used for this analysis are drawn from birth records spanning 2008 to 2019, with individual births as the unit of observation.

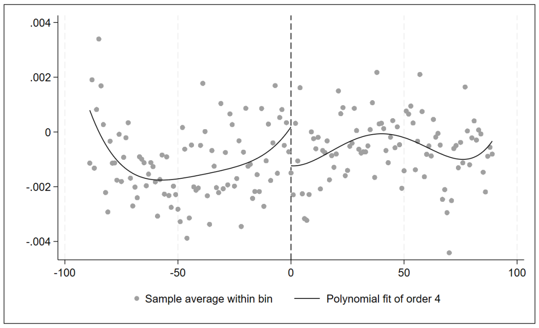

(a) Low birth weight (very)

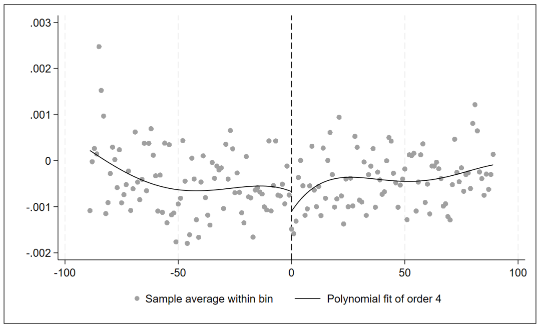

(b) Low birth weight (extreme)

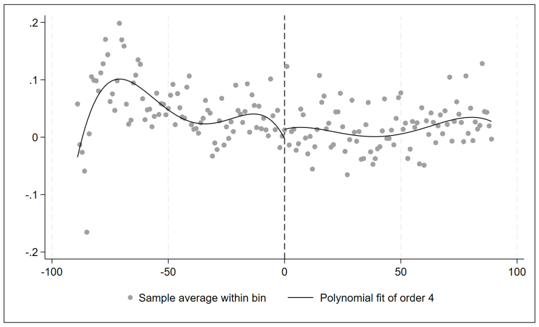

(c) Gestational length

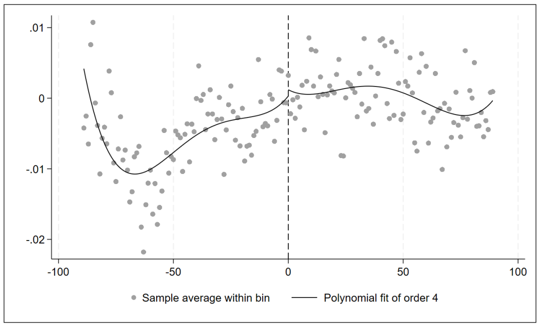

(d) Pre-term pregnancy

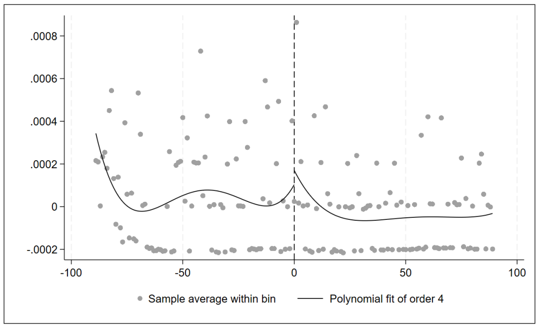

(e) Post-term pregnancy

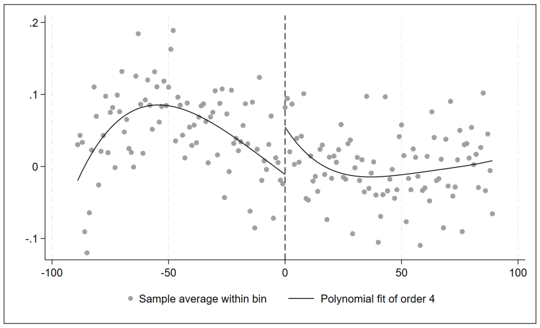

(f) Height

Figure A6: Tax effect on supplementary outcomes. *Notes*: The graph displays the de-meaned and seasonally adjusted residuals for various outcomes related to gestational age, low birth weight and height: (a) Low birth weight (very), (b) Low birth weight (extreme), (c) gestational length, (d) pre-term pregnancy, (e) post-term pregnancy and (f) height. To estimate the impact of the tax rate increase on January 1st, 2011, residuals are used to generate a scatter plot. The date at entering the third trimester relative to the cut-off date is used as the exposure of interest. This plot is then utilized to make separate polynomial predictions for both 90 days before and after the tax rate change while controlling for sociodemographic characteristics. The vertical line refers to January 1st 2011. Data used for this analysis are drawn from birth records spanning 2008 to 2019, with individual births as the unit of observation.

B Influence of Maternal Characteristics on Birth Outcomes

Table B1: Estimation Results for Birth Weight Outcomes by Mother's Age

	Total<br>(1)	Aged 15 to 19<br>(2)	Aged 20 to 29<br>(3)	Aged 30 to 44<br>(4)
Panel A: Weight in grams				
RD Estimate	10.2745**	11.3825	3.0728	18.5971*
	(5.2309)	(11.3248)	(5.6316)	(10.3649)
Mean (left)	3164.2	3101.76	3169.08	3196.09
Observations	822,244	146,143	454,020	222,081
Bias Corrected Effect	12.6030	15.1878	5.3191	23.2581
Robust Std. Error	5.7620	13.2319	6.4018	11.3378
Panel B: Low birth weight				
RD Estimate	-0.0036	-0.0045	0.0003	-0.0034
	(0.0029)	(0.0071)	(0.0027)	(0.0046)
Mean (left)	.07	.08	.06	.07
Observations	822,244	146,143	454,020	222,081
Bias Corrected Effect	-0.0047	-0.0066	-0.0002	-0.0053
Robust Std. Error	0.0033	0.0084	0.0033	0.0051
Panel C: Normal birth weight				
RD Estimate	0.0016	0.0025	-0.0002	0.0034
	(0.0033)	(0.0076)	(0.0036)	(0.0061)
Mean (left)	.9	.9	.9	.88
Observations	822,244	146,143	454,020	222,081
Bias Corrected Effect	0.0025	0.0038	-0.0001	0.0048
Robust Std. Error	0.0039	0.0092	0.0044	0.0072
Panel D: Fetal macrosomia				
RD Estimate	0.0016	0.0016	0.0010	0.0020
	(0.0020)	(0.0033)	(0.0022)	(0.0043)
Mean (left)	.03	.02	.03	.05
Observations	822,244	146,143	454,020	222,081
Bias Corrected Effect	0.0023	0.0024	0.0013	0.0033
Robust Std. Error	0.0023	0.0040	0.0026	0.0050
Week & Mun. & Year FE	Yes	Yes	Yes	Yes
Unemployment rate	Yes	Yes	Yes	Yes
Sociodem. Charact.	Yes	Yes	Yes	Yes

Notes: This table estimates the effect of the tax on weight related outcomes contingent on maternal age cohorts. Outcomes include (A) birth weight in grams, (B) low birth weight, (C) normal birth weight and (D) fetal macrosomia. Impact is estimated using a RD methodology with time as the running variable and the Tax serving as the treatment dummy. Data used for this analysis are drawn from birth records spanning 2008 to 2019, with individual births as the unit of observation. Each column corresponds to a distinct regression using January 1st as the cutoff and assigning treatment based on exposure determined based on the date at entering the third trimester (3rd tri). Residuals of the outcome variables are used as dependent variables. All estimations consist of a local linear regression of a second degree polynomial with triangular kernel weights and a 90-day bandwidth, estimated using rdrobust in Stata. * .10 ** .05 *** .01 sig. levels. Robust standard errors in parentheses.

Table B2: Estimation Results for Birth Weight Outcomes by Mother's Educational Attainment

	Total<br>(1)	Less than high school<br>(2)	High school<br>(3)	College or more<br>(4)
Panel A: Weight in grams				
RD Estimate	10.2745**	10.8043*	12.8304	-12.7055
	(5.2309)	(6.0766)	(9.4483)	(11.9237)
Mean (left)	3164.2	3164.4	3163.75	3164.15
Observations	822,244	509,354	210,113	102,777
Bias Corrected Effect	12.6030	13.5102	16.3936	-13.3570
Robust Std. Error	5.7620	6.7156	10.8268	14.3340
Panel B: Low birth weight				
RD Estimate	-0.0036	-0.0040	-0.0078	0.0089
	(0.0029)	(0.0032)	(0.0055)	(0.0061)
Mean (left)	.07	.07	.07	.07
Observations	822,244	509,354	210,113	102,777
Bias Corrected Effect	-0.0047	-0.0049	-0.0100	0.0087
Robust Std. Error	0.0033	0.0037	0.0063	0.0073
Panel C: Normal birth weight				
RD Estimate	0.0016	0.0028	0.0096	-0.0158**
	(0.0033)	(0.0039)	(0.0065)	(0.0080)
Mean (left)	.9	.9	.9	.9
Observations	822,244	509,354	210,113	102,777
Bias Corrected Effect	0.0025	0.0031	0.0122	-0.0165
Robust Std. Error	0.0039	0.0047	0.0074	0.0096
Panel D: Fetal macrosomia				
RD Estimate	0.0016	0.0017	-0.0016	0.0073
	(0.0020)	(0.0026)	(0.0029)	(0.0048)
Mean (left)	.03	.04	.03	.03
Observations	822,244	509,354	210,113	102,777
Bias Corrected Effect	0.0023	0.0026	-0.0015	0.0080
Robust Std. Error	0.0023	0.0029	0.0035	0.0057
Week & Mun. & Year FE	Yes	Yes	Yes	Yes
Unemployment rate	Yes	Yes	Yes	Yes
Sociodem. Charact.	Yes	Yes	Yes	Yes

Notes: This table estimates the effect of the tax on weight related outcomes contingent on maternal educational attainment. Outcomes include (A) birth weight in grams, (B) low birth weight, (C) normal birth weight and (D) fetal macrosomia. Impact is estimated using a RD methodology with time as the running variable and the Tax serving as the treatment dummy. Data used for this analysis are drawn from birth records spanning 2008 to 2019, with individual births as the unit of observation. Each column corresponds to a distinct regression using January 1st as the cutoff and assigning treatment based on exposure determined based on the date at entering the third trimester (3rd tri). Residuals of the outcome variables are used as dependent variables. All estimations consist of a local linear regression of a second degree polynomial with triangular kernel weights and a 90-day bandwidth, estimated using rdrobust in Stata. * .10 ** .05 *** .01 sig. levels. Robust standard errors in parentheses.

Table B3: Estimation Results for Birth Weight Outcomes by Mother's Live Birth Order

	Total<br>(1)	First birth<br>(2)	Second birth<br>(3)	Third birth or more<br>(4)
Panel A: Weight in grams				
RD Estimate	10.2745**	5.4341	1.6755	14.5408
	(5.2309)	(8.0912)	(7.4847)	(9.0975)
Mean (left)	3164.2	3118.16	3176.77	3204.57
Observations	822,244	303,340	240,632	278,272
Bias Corrected Effect	12.6030	8.0485	4.3960	17.9930
Robust Std. Error	5.7620	9.4742	8.5687	10.3628
Panel B: Low birth weight				
RD Estimate	-0.0036	0.0018	-0.0061	-0.0034
	(0.0029)	(0.0047)	(0.0047)	(0.0038)
Mean (left)	.07	.08	.06	.07
Observations	822,244	303,340	240,632	278,272
Bias Corrected Effect	-0.0047	0.0004	-0.0078	-0.0048
Robust Std. Error	0.0033	0.0055	0.0054	0.0043
Panel C: Normal birth weight				
RD Estimate	0.0016	-0.0026	0.0024	0.0039
	(0.0033)	(0.0052)	(0.0051)	(0.0049)
Mean (left)	.9	.9	.9	.89
Observations	822,244	303,340	240,632	278,272
Bias Corrected Effect	0.0025	-0.0016	0.0034	0.0045
Robust Std. Error	0.0039	0.0063	0.0061	0.0059
Panel D: Fetal macrosomia				
RD Estimate	0.0016	0.0006	0.0019	0.0004
	(0.0020)	(0.0025)	(0.0030)	(0.0034)
Mean (left)	.03	.02	.04	.05
Observations	822,244	303,340	240,632	278,272
Bias Corrected Effect	0.0023	0.0012	0.0027	0.0006
Robust Std. Error	0.0023	0.0030	0.0035	0.0041
Week & Mun. & Year FE	Yes	Yes	Yes	Yes
Unemployment rate	Yes	Yes	Yes	Yes
Sociodem. Charact.	Yes	Yes	Yes	Yes

Notes: This table estimates the effect of the tax on weight related outcomes contingent on the mother's live birth order. Outcomes include (A) birth weight in grams, (B) low birth weight, (C) normal birth weight and (D) fetal macrosomia. Impact is estimated using a RD methodology with time as the running variable and the Tax serving as the treatment dummy. Data used for this analysis are drawn from birth records spanning 2008 to 2019, with individual births as the unit of observation. Each column corresponds to a distinct regression using January 1st as the cutoff and assigning treatment based on exposure determined based on the date at entering the third trimester (3rd tri). All estimations consist of a local linear regression of a second degree polynomial with triangular kernel weights and a 90-day bandwidth, estimated using rdrobust in Stata. * .10 ** .05 *** .01 sig. levels. Robust standard errors in parentheses.

Table B4: Estimation Results for Birth Weight Outcomes by Mother's Marital Status

	Total (1)	Single (2)	Not single (3)
Panel A: Weight in grams			
RD Estimate	10.2745**	7.7265	9.5026*
	(5.2309)	(16.0379)	(5.1526)
Mean (left)	3164.2	3121.65	3169.16
Observations	822,244	84,957	737,287
Bias Corrected Effect	12.6030	13.8314	n.a.
Robust Std. Error	5.7620	18.4875	5.6385
Panel B: Low birth weight			
RD Estimate	-0.0036	0.0075	-0.0042
	(0.0029)	(0.0087)	(0.0029)
Mean (left)	.07	.08	.07
Observations	822,244	84,957	737,287
Bias Corrected Effect	-0.0047	0.0057	n.a.
Robust Std. Error	0.0033	0.0104	0.0033
Panel C: Normal birth weight			
RD Estimate	0.0016	-0.0031	0.0020
	(0.0033)	(0.0106)	(0.0033)
Mean (left)	.9	.89	.9
Observations	822,244	84,957	737,287
Bias Corrected Effect	0.0025	-0.0000	n.a.
Robust Std. Error	0.0039	0.0126	0.0039
Panel D: Fetal macrosomia			
RD Estimate	0.0016	-0.0037	0.0021
	(0.0020)	(0.0051)	(0.0020)
Mean (left)	.03	.03	.04
Observations	822,244	84,957	737,287
Bias Corrected Effect	0.0023	-0.0045	n.a.
Robust Std. Error	0.0023	0.0062	0.0024
Week & Mun. & Year FE	Yes	Yes	Yes
Unemployment rate	Yes	Yes	Yes
Sociodem. Charact.	Yes	Yes	Yes

Notes: This table estimates the effect of the tax on weight related outcomes contingent on mother's marital status. Outcomes include (A) birth weight in grams, (B) low birth weight, (C) normal birth weight and (D) fetal macrosomia. Impact is estimated using a RD methodology with time as the running variable and the Tax serving as the treatment dummy. Data used for this analysis are drawn from birth records spanning 2008 to 2019, with individual births as the unit of observation. Each column corresponds to a distinct regression using January 1st as the cutoff and assigning treatment based on exposure determined based on the date at entering the third trimester (3rd tri). Residuals of the outcome variables are used as dependent variables. All estimations consist of a local linear regression of a second degree polynomial with triangular kernel weights and a 90-day bandwidth, estimated using rdrobust in Stata. The bias-corrected estimate could not be computed due to insufficient bandwidth or collinearity in the data for some estimations. In these cases, the cells display "n.a." instead of a value, indicating that the estimate is not available. * .10 ** .05 *** .01 sig. levels. Robust standard errors in parentheses.

C Robustness Checks

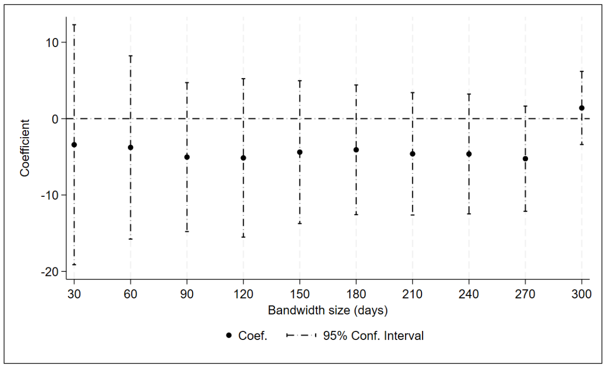

(a) Birth weight in grams

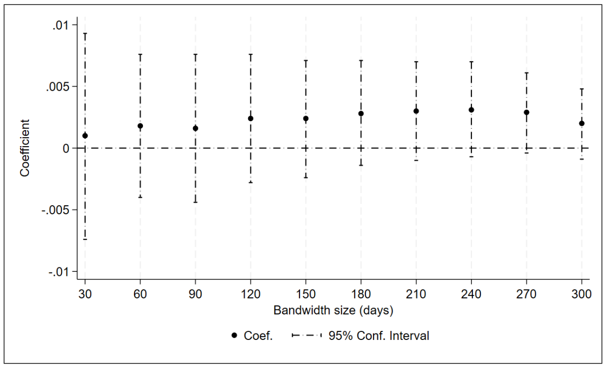

(b) Low birth weight

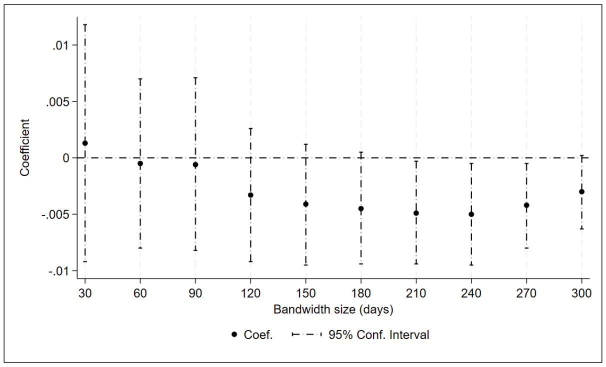

(c) Normal birth weight

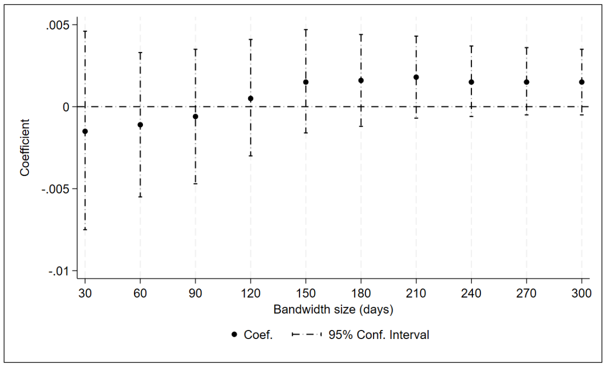

(d) Fetal macrosomia

Figure C1: Effect of the November 19, 2010 announcement date on birth weight. *Notes:* This figure displays point estimates for the effect of the tax on (a) birth weight in grams, (b) low birth weight, (c) normal birth weight and (d) fetal macrosomia using a RD methodology with time as the running variable and the announcement date for the tax increase serving as the treatment dummy. The tax was announced on November 19, 2010, and the introduction date was January 1, 2011. Each data point comes from a different regression using November 19 of 2010 as the cutoff assigning treatment based on exposure determined based on the date at entering the first third (3rd tri). Each data point uses a bandwidth of 90 days before and after the tax rate change. Data used for this analysis are drawn from birth records spanning 2008 to 2019, with individual births as the unit of observation. Each regression included is based on a specification using de-meaned and seasonally adjusted residuals and includes sociodemographic characteristics as controls.

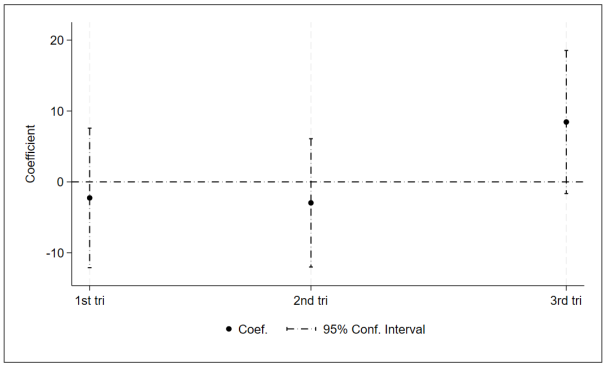

(a) Birth weight in grams

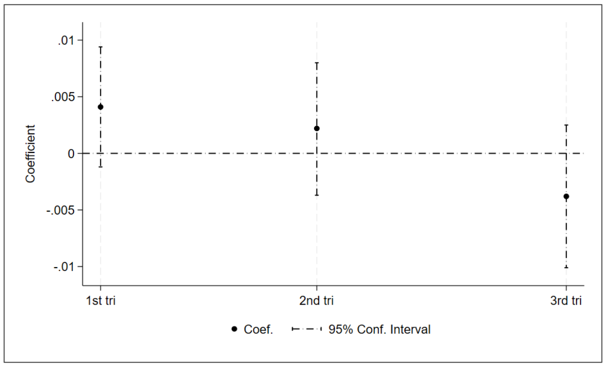

(b) Low birth weight

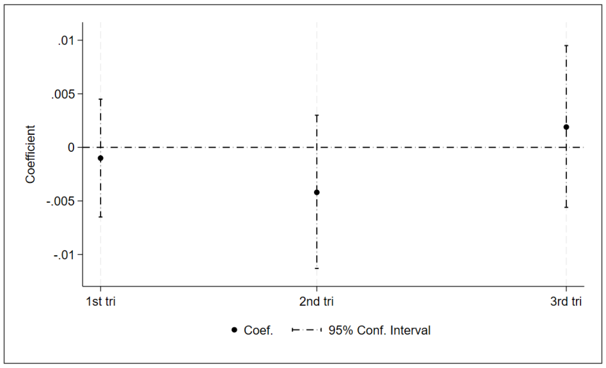

(c) Normal birth weight

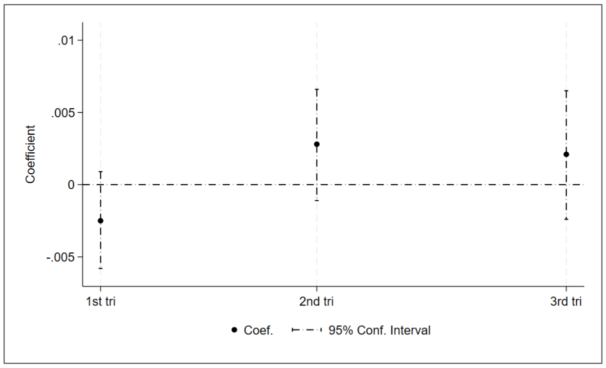

(d) Fetal Macrosomia

Figure C2: Tax effect on Birth Weight by Trimester of Gestation Including Potential Data Entry Errors. *Notes:* This figure displays point estimates for the effect of the tax on (a) birth weight in grams, (b) low birth weight, (c) normal birth weight and (d) fetal macrosomia using a RD methodology with time as the running variable and the Tax serving as the treatment dummy. This analysis includes all observations regardless of birth weight, including those below 500 grams and above 7,000 grams, which may reflect data entry errors. Each data point originates from a distinct regression using January 1st as the cutoff and assigning treatment based on exposure determined at the date entering the first trimester (1st tri), the date at entering second trimester (2nd tri), and the date at entering third trimester (3rd tri). Each data point uses a bandwidth of 90 days before and after the tax rate change. Data used for this analysis are drawn from birth records spanning 2008 to 2019, with individual births as the unit of observation. Each regression included is based on a specification using de-meaned and seasonally adjusted residuals and includes sociodemographic characteristics as controls.

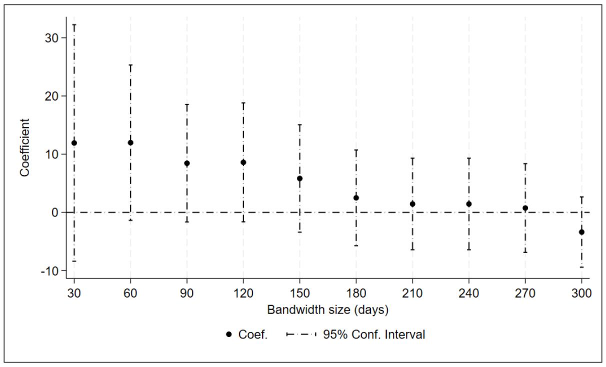

(a) Birth weight in grams

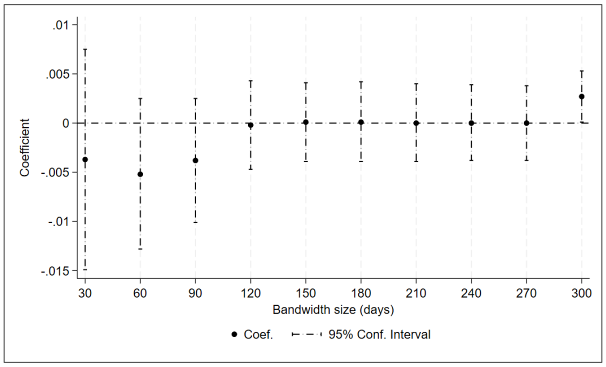

(b) Low birth weight

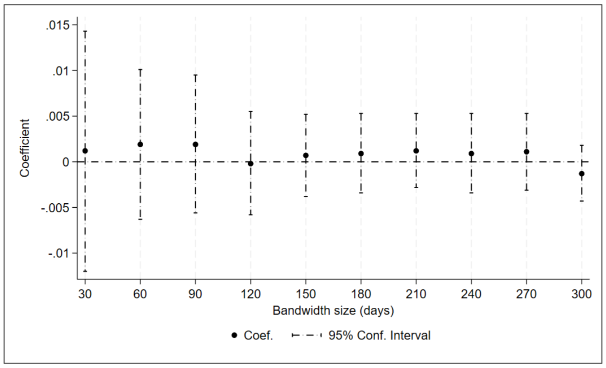

(c) Normal birth weight

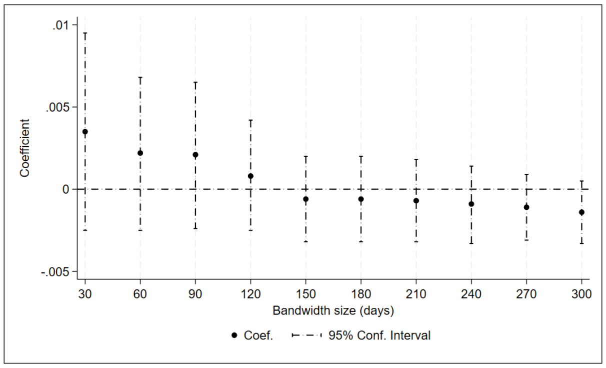

(d) Fetal Macrosomia

Figure C3: Tax effect on birth weight by bandwidth Including Potential Data Entry Errors. *Notes:* This figure displays point estimates for the effect of the tax on (a) birth weight in grams, (b) low birth weight, (c) normal birth weight and (d) fetal macrosomia using a RD methodology with time as the running variable and the Tax serving as the treatment dummy. This analysis includes all observations regardless of birth weight, including those below 500 grams and above 7,000 grams, which may reflect data entry errors. Each data point originates from a distinct regression using January 1st as the cutoff and assigning treatment based on exposure determined based on the date at entering the third trimester (3rd tri) but uses a different bandwidth of days before and after the tax rate change. Data used for this analysis are drawn from birth records spanning 2008 to 2019, with individual births as the unit of observation. Each regression included is based on a specification using de-meaned and seasonally adjusted residuals and includes sociodemographic characteristics as controls.

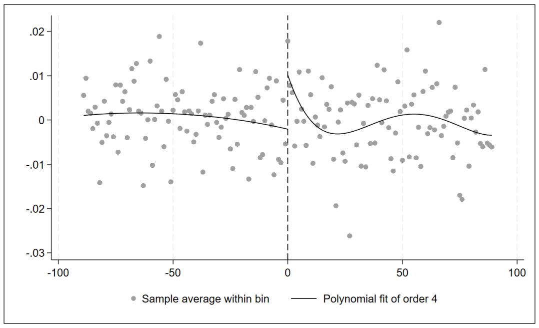

(a) Birth sex being female

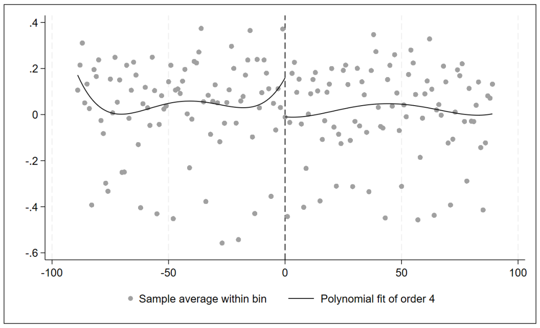

(b) Age of the mother

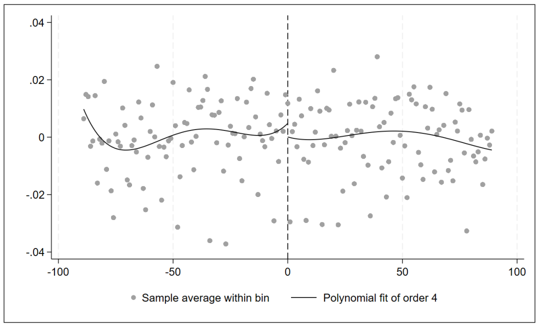

(c) Marriage status

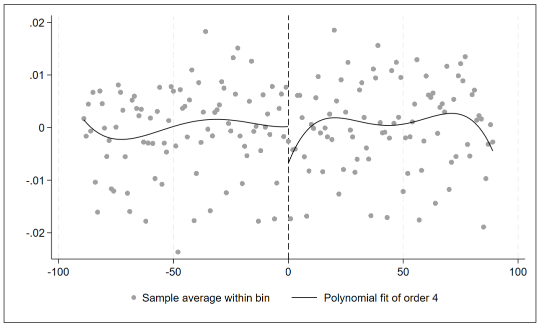

(d) Working Status

Figure C4: Effects on unrelated or placebo outcomes. Notes: The graph displays the de-meaned and seasonally adjusted residuals for (a) Birth sex being female, (b) age of the mother, (c) marriage status, (d) working status. To estimate the impact of the tax rate increase on January 1st, 2011, residuals are used to generate a scatter plot. The date at entering the third trimester relative to the cut-off date is used as the exposure of interest. This plot is then utilized to make separate polynomial predictions for both 90 days before and after the tax rate change while controlling for sociodemographic characteristics. The vertical line refers to January 1st 2011. Data used for this analysis are drawn from birth records spanning 2008 to 2019, with individual births as the unit of observation.

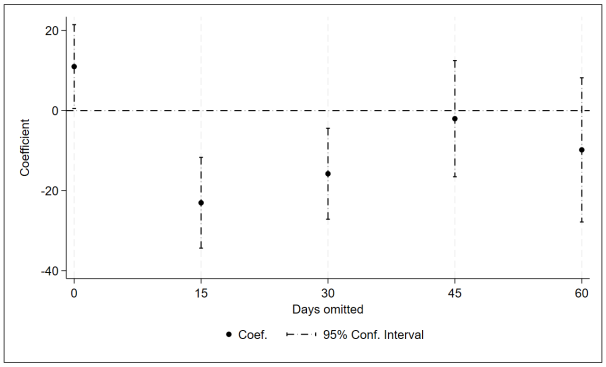

(a) Birth weight in grams

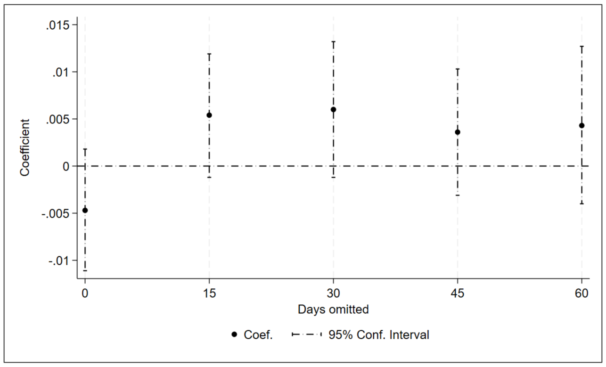

(b) Low birth weight

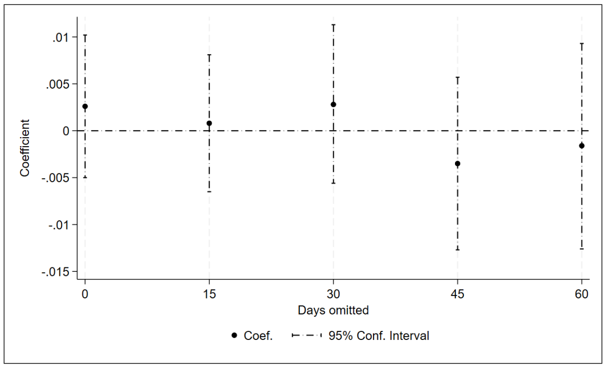

(c) Normal birth weight

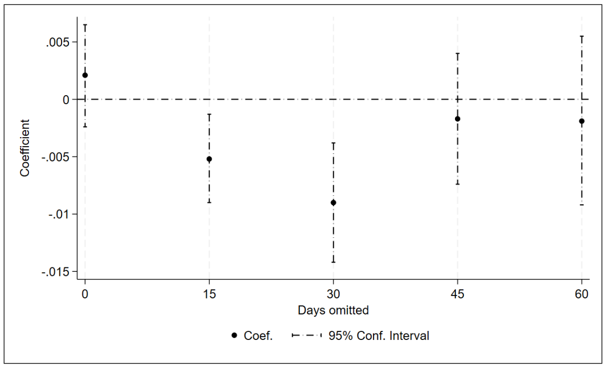

(d) Fetal Macrosomia

Figure C5: Results excluding observations centered on the cut-off. Notes: This figure displays point estimates for the effect of the tax on (a) birth weight in grams, (b) low birth weight, (c) normal birth weight and (d) fetal macrosomia using a RD methodology with time as the running variable and the Tax serving as the treatment dummy. Each data point originates from a distinct regression using January 1st as the cutoff and assigning treatment based on exposure determined based on the date at entering the third trimester (3rd tri), starting from a bandwidth of 90 days before and after the tax rate change while excluding a set number of days centered around the cut-off date of January 1st, 2011: 15, 30, 45 and 60 days. Data used for this analysis are drawn from birth records spanning 2008 to 2019, with individual births as the unit of observation. Each regression included is based on a specification using de-meaned and seasonally adjusted residuals that partial out week of the year dummies, municipality fixed effects, unemployment rate, and sociodemographic characteristics.

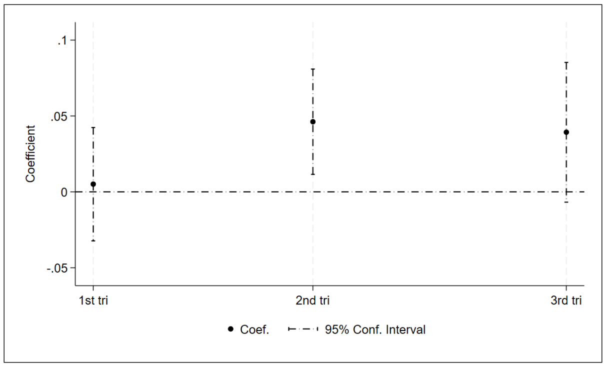

(a) Gestational length

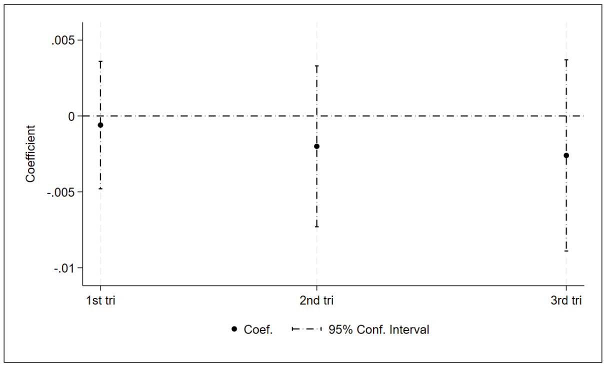

(b) Pre-term pregnancy

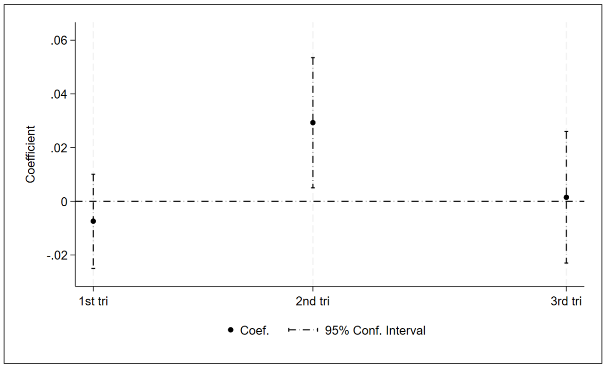

(c) Silverman score

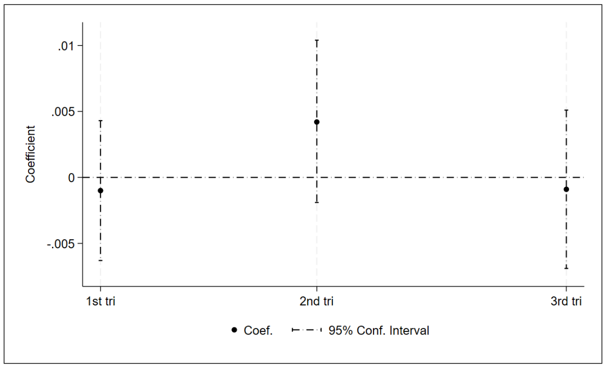

(d) Any respiratory problems

Figure C6: Trimester-Specific Tax Effect on gestational length and respiratory problems *Notes:* This figure displays point estimates for the effect of the tax on (a) gestational length (b) pre-term pregnancy, (c) Silverman score and (d) any respiratory problems using a RD methodology with time as the running variable and the Tax serving as the treatment dummy. Each data point originates from a distinct regression using January 1st as the cutoff and assigning treatment based on exposure determined at the date entering the first trimester (1st tri), the date at entering second trimester (2nd tri), and the date at entering third trimester (3rd tri). Each data point uses a bandwidth of 90 days before and after the tax rate change. Data used for this analysis are drawn from birth records spanning 2008 to 2019, with individual births as the unit of observation. Each regression included is based on a specification using de-meaned and seasonally adjusted residuals and includes sociodemographic characteristics as controls.

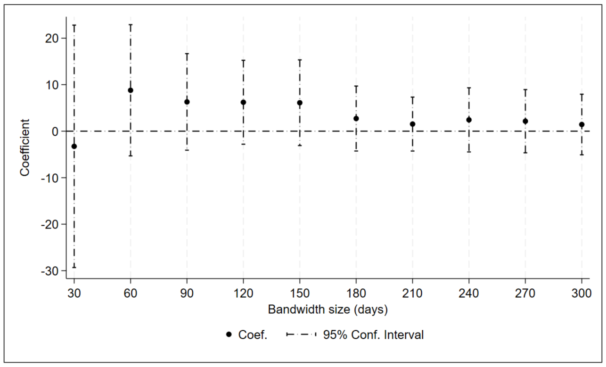

(a) Birth weight in grams

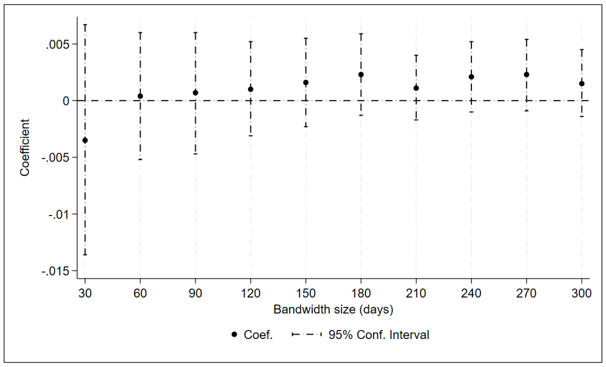

(b) Low birth weight

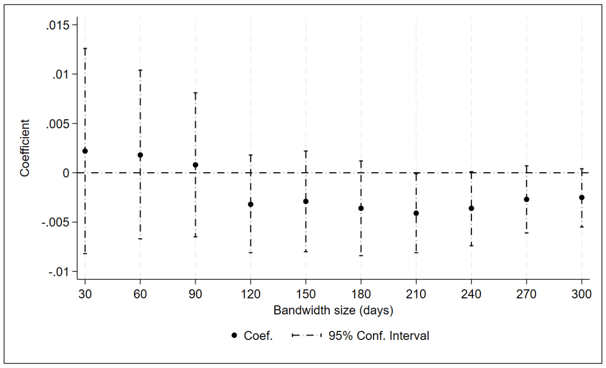

(c) Normal birth weight

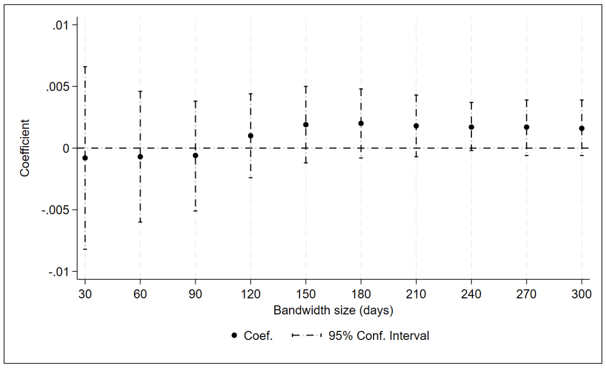

(d) Fetal Macrosomia

Figure C7: Tax effect on birth weight by bandwidth assigning treatment based on the time of birth. *Notes:* This figure displays point estimates for the effect of the tax on (a) birth weight in grams, (b) low birth weight, (c) normal birth weight and (d) fetal macrosomia using a RD methodology with time as the running variable and the Tax serving as the treatment dummy. Each data point comes from a different regression using January 1st as the cutoff. This specification assigns treatment based on entry into the time of birth, instead of entry into the third trimester. Treatment is determined based on the date of birth but using a different bandwidth of days before and after the tax rate change. Data used for this analysis are drawn from birth records spanning 2008 to 2019, with individual births as the unit of observation. Each regression included is based on a specification using de-meaned and seasonally adjusted residuals and includes sociodemographic characteristics as controls.

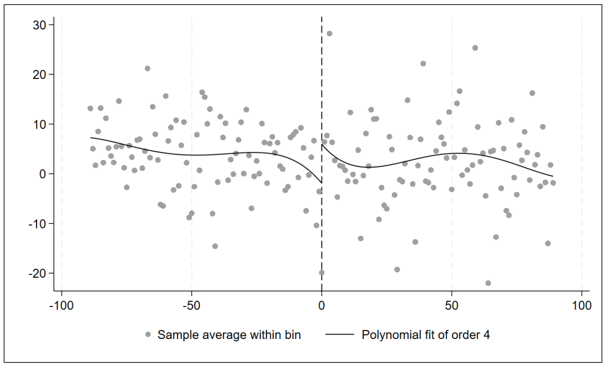

(a) Birth weight in grams

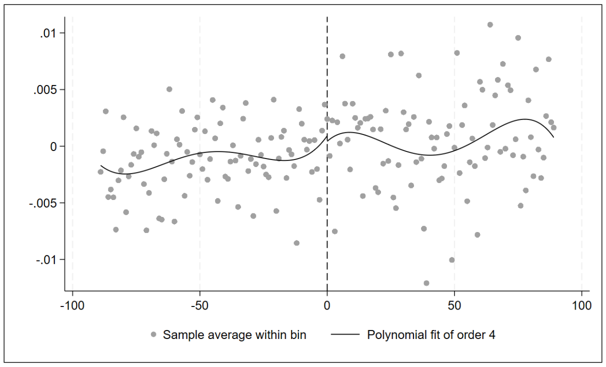

(b) Low birth weight

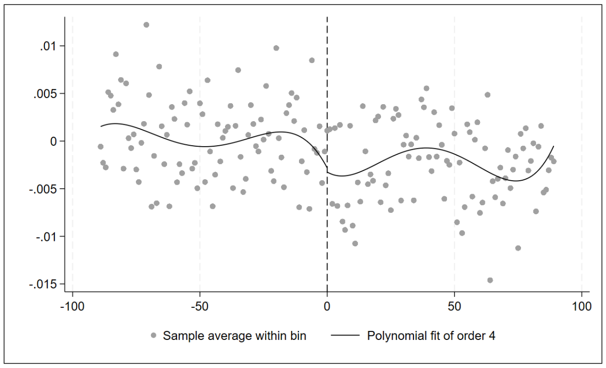

(c) Normal birth weight

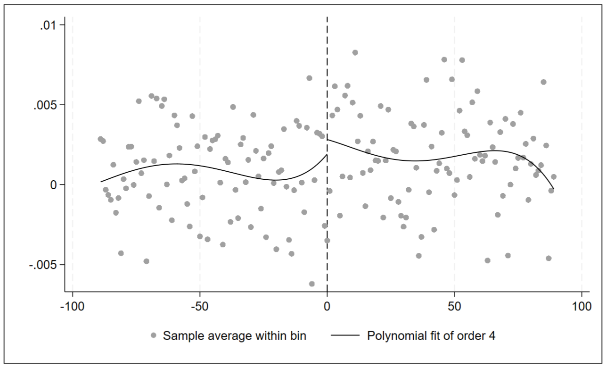

(d) Fetal macrosomia

Figure C8: Tax effect using the date of birth as the measure of exposure. Notes: The graph displays the de-meaned and seasonally adjusted residuals for various outcomes related to birth weight: (a) birth weight in grams, (b) Low birth weight, (c) normal birth weight and (d) fetal macrosomia. To estimate the impact of the tax rate increase on January 1st, 2011, residuals are used to generate a scatter plot. This specification assigns treatment based on entry into the time of birth, instead of entry into the third trimester. This plot is then utilized to make separate polynomial predictions for both 90 days before and after the tax rate change. The vertical line refers to January 1st 2011. Data used for this analysis are drawn from birth records spanning 2008 to 2019, with individual births as the unit of observation. By examining the change in level at this time discontinuity, we can determine an estimate of how much the tax has influenced the corresponding outcome.

D Exposure in Ongoing Pregnancies at the Time of Implementation

To further investigate the effects of the tax policy, we employ a specification that accounts for varying exposure durations during pregnancy. The sample under analysis encompasses births from the inception of the tax policy on January 1st, 2011, until October 9, 2011, spanning 40 weeks, the typical duration of pregnancy. The model distinguishes between one trimester, three trimesters, and two trimesters of exposure to the tax, with the latter serving as the reference category. Specifically, we estimate the following equation:

$$\hat{Y}_{it} = \alpha + \beta \text{One Trim. of Exposure}_{it} + \gamma \text{Three Trim. of Exposure}_{it} + X_{it} + \epsilon_{it} \quad (3)$$

where $\hat{Y}_{it}$ represents the de-meaned and seasonally adjusted residuals of the outcome variable; α represents a constant term; One Trim. of Exposure$_{it}$ takes the value of 1 if birth happened between January 1st and March 26th and 0 otherwise and Three Trim. of Exposure$_{it}$ takes the value of 1 if the baby was born between July 10th to October 9th and 0 otherwise. Two Trim. of Exposure$_{it}$, the omitted category, takes the value of 1 if the baby was born between on all singleton live births in 27th and July 9th, and 0 otherwise; X_{it} is a vector of individual control variables; M_i represent municipality fixed effects; and ϵ_{it} is the error term.

All comparisons are made using two trimesters of exposure as the category of reference. Our analysis found that pregnancies exposed to the tax introduction for one trimester showed increased weight ranging from 5 to 6 grams, no improvements are observed for three trimesters of exposure. Conversely, we observe improvements for low birth weight among births exposed to the longest duration within the sample, that is, three trimesters of exposure. Additionally, we note increases in macrosomia accompanied by decreasing prevalence of normal weight for those births exposed for one trimester, indicating the increase in average weight is shifting the distribution towards higher weights. The results for weight in grams, normal weight, and macrosomia align with the analysis of birth weight by bandwidth from the regression discontinuity analysis, yet the analysis for low birth weight offers additional insights. While the regression discontinuity analysis reveals reductions in low birth weight when comparing births exposed to the tax policy versus those that were not, when comparing women that were already pregnant at the time of introduction of the tax, we find the higher gains in terms of preventing low birth weight correspond to those with greater exposure duration.

Table D1: Estimation Results for Birth Weight Outcomes of Women already Pregnant

	(1)	(2)	(3)
Panel A: Weight in grams			
One Trim. of Exposure	4.7174***	4.9840***	6.2835***
	(1.2409)	(1.2858)	(1.2775)
Three Trim. of Exposure	1.8034	1.8034	1.8034
	(1.4219)	(1.4127)	(1.4546)
Mean	3142.3406	3142.3406	3142.3406
Observations	1,360,882	1,360,882	1,360,882
Panel B: Low birth weight			
One Trim. of Exposure	0.0003	0.0001	-0.0002
	(0.0006)	(0.0006)	(0.0006)
Three Trim. of Exposure	-0.0011*	-0.0011**	-0.0011
	(0.0006)	(0.0006)	(0.0006)
Mean	.0759	.0759	.0759
Observations	1,360,882	1,360,882	1,360,882
Panel C: Normal birth weight			
One Trim. of Exposure	-0.0026***	-0.0024***	-0.0023***
	(0.0006)	(0.0006)	(0.0006)
Three Trim. of Exposure	0.0011	0.0011*	0.0011
	(0.0007)	(0.0007)	(0.0007)
Mean	.8925	.8925	.8925
Observations	1,360,882	1,360,882	1,360,882
Panel D: Fetal macrosomia			
One Trim. of Exposure	0.0022***	0.0023***	0.0025***
	(0.0004)	(0.0004)	(0.0004)
Three Trim. of Exposure	-0.0001	-0.0001	-0.0001
	(0.0005)	(0.0005)	(0.0005)
Mean	.0316	.0316	.0316
Observations	1,360,882	1,360,882	1,360,882
Week & Mun. & Year FE	Yes	Yes	Yes
Unemployment rate	No	Yes	Yes
Sociodem. Charact.	No	No	Yes

Notes: This table estimates the effect of the tax on weight-related outcomes, including (a) birth weight in grams, (b) low birth weight, (c) normal birth weight, and (d) fetal macrosomia, for women who were already pregnant when the tax was introduced. We focus on women who gave birth from January 1, 2011, to October 9, 2011, which covers 40 weeks of pregnancy. We categorize births based on the number of trimesters of exposure to the tax: babies born from January 1 to March 26 had one trimester of exposure; those born between March 27 and July 9 had two trimesters of exposure; and those born from July 10 to October 9 had three trimesters of exposure. Each column represents a distinct regression, with two trimesters of exposure as the omitted category. Each regression included is based on a specification using de-meaned and seasonally adjusted residuals. Column (1) displays results after partialling out municipality, week of the year fixed effects, and year fixed effects. In column (2), the model additionally accounts for the unemployment rate, while column (3) incorporates sociodemographic characteristics as covariates. All estimations consist of a linear regression estimated Stata. * .10 ** .05 *** .01 sig. levels. Robust standard errors in parentheses.
